# Molecular profiling of resident and infiltrating mononuclear phagocytes during rapid adult retinal degeneration using single-cell RNA sequencing

**DOI:** 10.1038/s41598-019-41141-0

**Published:** 2019-03-19

**Authors:** Kaitryn E. Ronning, Sarah J. Karlen, Eric B. Miller, Marie E. Burns

**Affiliations:** 1 Center for Neuroscience, University of California Davis, Davis, CA 95618 USA; 2 Department of Cell Biology and Human Anatomy, University of California Davis, Davis, CA 95616 USA; 3 Department Ophthalmology & Vision Science, University of California Davis, Davis, CA 95616 USA

## Abstract

Neuroinflammation commonly accompanies neurodegeneration, but the specific roles of resident and infiltrating immune cells during degeneration remains controversial. Much of the difficulty in assessing myeloid cell-specific functions during disease progression arises from the inability to clearly distinguish between activated microglia and bone marrow-derived monocytes and macrophages in various stages of differentiation and activation within the central nervous system. Using an inducible model of photoreceptor cell death, we investigated the prevalence of infiltrating monocytes and macrophage subpopulations after the initiation of degeneration in the mouse retina. *In vivo* retinal imaging revealed infiltration of CCR2^+^ leukocytes across retinal vessels and into the parenchyma within 48 hours of photoreceptor degeneration. Immunohistochemistry and flow cytometry confirmed and characterized these leukocytes as CD11b^+^CD45^+^ cells. Single-cell mRNA sequencing of the entire CD11b^+^CD45^+^ population revealed the presence of resting microglia, activated microglia, monocytes, and macrophages as well as 12 distinct subpopulations within these four major cell classes. Our results demonstrate a previously immeasurable degree of molecular heterogeneity in the innate immune response to cell-autonomous degeneration within the central nervous system and highlight the necessity of unbiased high-throughput and high-dimensional molecular techniques like scRNAseq to understand the complex and changing landscape of immune responders during disease progression.

## Introduction

Although the central nervous system (CNS) was once considered entirely immune-privileged, there is growing evidence that interplay between neurons, glia, and the immune system are vital to healthy synaptic function^1^. Microglia, the resident macrophages of the CNS, are essential for synaptic homeostasis and plasticity and have been implicated in many neurodevelopmental and neurodegenerative diseases^2^. In contrast, infiltration of peripheral leukocytes into the CNS is considered rare and to primarily follow physical trauma or infection^3,4^. In the retina, infiltrating monocytes are associated with chemical or photolytic injury of the retinal pigment epithelium (RPE), which contributes to the blood-retinal barrier^5–9^. The differentiation of monocytes into microglia-like macrophages within the retina further challenges the ability to discern functional differences, if any, between these two distinct populations^8,10^. While there are some useful expression markers to differentiate between immune cell types, particularly when used in combination for immunohistochemistry^11^ or flow cytometry^8^, most transcriptomic and proteomic analyses are applied to entire populations, inherently averaging across subclasses and obfuscating cellular diversity.

Recent advances in single-cell RNA sequencing (scRNAseq) provide an exciting opportunity to understand the unique roles of individual cells in a high-throughput platform. Here we paired scRNAseq with a well-characterized inducible model of photoreceptor degeneration, the *Arr1*^*−/−*^ mouse^12^. Arrestin1, which is also known as retinal S-antigen (gene ID *Sag*, here refered to as *Arr1*), is a photoreceptor-specific cytosolic protein that deactivates photoexcited rhodopsin, shutting down the rod phototransduction cascade^13^. In the absence of Arr1, rods become extraordinarily sensitive^12^ and degenerate upon light exposure^14–16,17^. Even relatively dim light causes rapid ultrastructural disruption of *Arr1*^*−/−*^ photoreceptor inner segments, swelling of neighboring glia (Müller cells), and activation and migration of microglia into the photoreceptor layer within 24 hours^18^. Because *Arr1* is expressed only in photoreceptors, the *Arr1*^*−/−*^ model presents a unique opportunity to study the heterogeneity of immune responders in a time-locked manner when a specific class of neuron begins to die.

Using *in vivo* imaging, flow cytometry, and scRNAseq, we here report profound differences in the inflammatory profiles, mitotic activity, and active phagocytosis of distinct subpopulations of microglia, monocytes, and monocyte-derived macrophages within 48 hours of the onset of rod degeneration. These results reveal a greater level of phenotypic variety than previously appreciated, adding to the complexity of understanding the role of immune cells, even at short times after the onset of neurodegeneration.

## Results

### Invasion of peripheral immune cells into the rapidly degenerating retina

**Figure 1:**
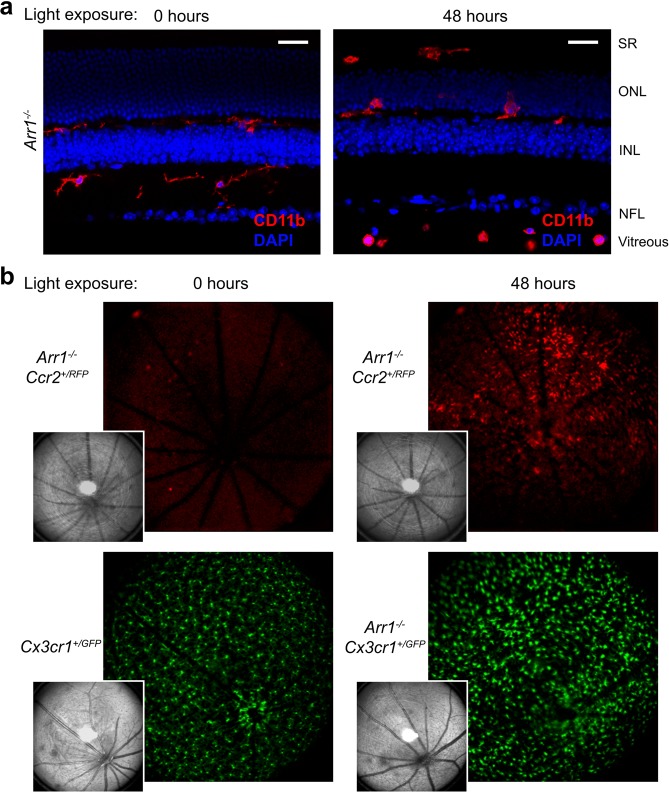
Immune cells respond to acute photoreceptor degeneration. (**a**) Immunohistochemical sections before and after onset of photoreceptor degeneration. After 48 hours of light exposure, CD11b^+^ cells that were round appeared in the vitreous and nerve fiber layer (NFL) near large caliber vessels while those that were ameboid were present in the subretinal space (SR) and photoreceptor layer (outer nuclear layer, ONL). Scale bars indicate 25 µm, INL = inner nuclear layer. (**b**) *In vivo* retinal imaging using scanning laser ophthalmoscopy. Cells expressing RFP driven by the CCR2 promoter (putative monocytes) appeared abruptly within 48 hours of light exposure (compare 0 to 48 hours exposure taken from the same mouse), while the number of GFP expressing resident microglia of changed little. Insets show corresponding reflectance images.

The *Arr1*^*−/−*^ mouse is a convenient, light-inducible model of widespread, cell-autonomous photoreceptor neurodegeneration^14,17^. Previous studies have shown that within 24 hours of light onset, microglia change morphology and migrate into the photoreceptor layer, and between 36 and 72 hours after light onset there is a dramatic increase in the number of Iba1^+^ cells present in the retina^18^. We first aimed to investigate the source of this increase in cell number using immunohistochemistry on *Arr1*^*−/−*^ retinas exposed to 48 hours of light. Sections of retina immunohistochemically stained for CD11b, a pan-myeloid cell marker, showed the presence of enlarged macrophage-like cells in and around the photoreceptors and subretinal space. Additionally, there were small round CD11b^+^ monocyte-like cells often visible at the vitreoretinal surface and retinal layers of light exposed mice that were never observed when the animals were maintained in darkness (Fig. 1a).

Monocyte extravasation into the retina has been implicated in other mouse models of retinal and RPE damage known to disrupt the blood-retina barrier (for example, see^6^). However, to our knowledge, leukocyte infiltration has not been previously known to occur in any models of cell-autonomous retinal neurodegeneration. To confirm the presence of infiltrating cells *in vivo*, we crossed *Arr1*^*−/−*^ mice with *Ccr2*^*RFP/RFP*^ knock-in mice that express RFP downstream of the *Ccr2* promoter. Using scanning laser ophthalmoscopy, we could detect very little RFP^+^ signal within the retinas of dark-reared *Arr1*^*−/−*^ *Ccr2*^*+/RFP*^ mice. However, when the same mouse was imaged after 48 hours of light exposure, many RFP^+^ cells were evident within the retinal parenchyma (Fig. 1b, red), consistent with monocyte infiltration. These cells were distinct from microglial cells, which showed a different morphology and pattern of distribution at this same time point *in vivo* (*Arr1*^*−/−*^ *Cx3CR1*^+*/GFP*^; Fig. 1b, green). These results suggest that there are multiple distinct populations of immune cells that rapidly respond at the onset of photoreceptor degeneration.

### Single cell profiling reveals four classes of CD11b^+^CD45^+^ cells

**Figure 2:**
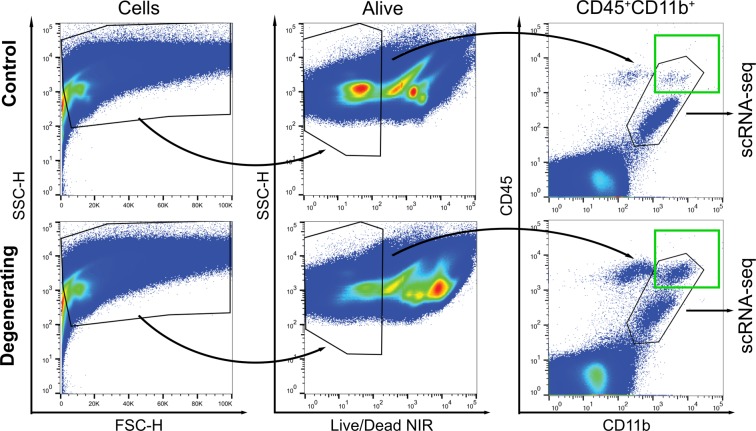
Immune response to photoreceptor degeneration can be identified and captured using FACS. After retinal dissociation, cells were detected using side scatter (SSC) and forward scatter height (FSC-H); cells that were alive (Live/Dead NIR low) and double positive for CD11b and CD45 were captured for single-cell mRNA sequencing. Green box indicates CD11b^+^CD45^high^ population, which was far larger in the degenerating sample. Black polygon encompasses cells captured for single-cell transcriptomics.

To more precisely identify resident and peripheral immune cell classes in the retina we used single-cell RNA sequencing. Retinas of healthy dark-reared control and degenerating (light-exposed) *Arr1*^*−/−*^ littermates were dissociated, and all live cells expressing both CD11b and CD45, which include both microglia and monocytes, were collected using FACS for single-cell RNA sequencing (Fig. 2, black polygon). Posthoc analysis of the collected cells showed a dramatic increase in the number of CD11b^+^ CD45^high^ cells (Fig. 2, green box), consistent with the invasion of peripheral cells seen by *in vivo* imaging. Interestingly, there was also an increase in the number of CD11b^−^ CD45^high^ cells during degeneration (Fig. 2); these cells were not captured for scRNAseq and will be the subject of future study.

**Figure 3:**
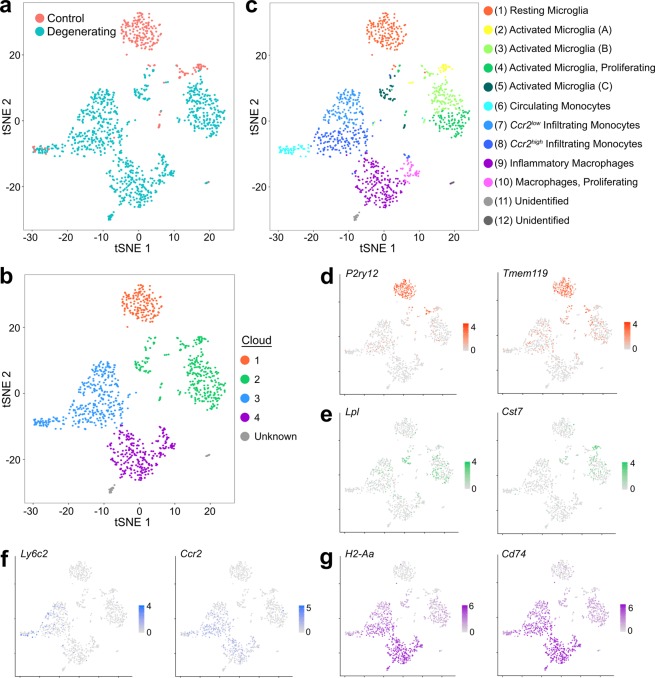
Identification of immune cells before and during retinal degeneration, displayed in tSNE plot of all cells, collapsed from a *k*-nearest neighbor comparison of all gene expression comprising the top 10 principal components. (**a**) Cells color-coded by sample identity, with immune cells from dark-reared control retinas in red and from degenerating retinas in blue. (**b**) Cells can be broadly grouped into 4 clouds, which correspond to the main 4 cell types: (1) resting microglia, (2) activated microglia, (3) monocytes, and (4) monocyte-derived macrophages. (**c**) Cells color-coded by cluster, identified by an unsupervised graph-based algorithm, and assigned cell identity using relative abundance of established markers. (**d**–**g**) Example marker gene expression of genes associated with resting microglia (**d**), activated microglia (**e**), monocytes (**f**), and monocyte-derived macrophages (**g**). Scales in (**d**–**g**) indicate relative expression (log TPM values), with grey indicating no expression and saturated color indicating highest expression.

Single-cell mRNA libraries of CD11b^+^CD45^+^ cells were generated and sequenced with stringent pre-processing and quality control cell filtering. The transcriptomes of all cells were compared using principal components analysis, and data were displayed in two dimensions using a *t*-distributed stochastic neighbor embedding (tSNE) plot where the relative position of each point reflects the genetic similarity of the cell to others in the population. The majority of immune cells in the dark-reared control sample (Fig. 3a, red) formed a well-defined cloud that did not overlap with cells from the degenerating retina. Instead three new qualitatively distinct clouds emerged in the degenerating sample (Fig. 3a, blue). This dissimilarity of control and degenerating samples from *Arr1*^*−/−*^ littermates indicates that the gene expression profiles themselves were not grossly affected by tissue processing, and suggests that these four clouds represent the presence of four distinct classes of CD11b^+^CD45^+^ cells (Fig. 3b).

To test whether the clouds indeed correspond to distinct cell classes, we queried the expression of numerous known or proposed microglia, monocytes, and macrophages markers across all cells (at least 15 markers per group). The expression of these markers was first examined graphically, by displaying the relative expression of each gene on a tSNE plot. Cloud #1 (Fig. 3b, orange), which was present only in control retina, expressed the highest levels of resting microglia markers, such as *P2ry12* and *Tmem119* (Fig. 3d); Cloud #2 (Fig. 3b, green) contained markers of microglial activation, such as *Lpl* and *Cst7* (Fig. 3e)^19,20^. Cloud #3 (Fig. 3b, blue) contained abundant monocyte markers such as *Ly6c2* and *Ccr2* (Fig. 3f), and in Cloud #4 (Fig. 3b, purple) markers of monocyte-derived macrophages like *H2-Aa* (an MHCII gene) and *Cd74* were most highly expressed (Fig. 3g)^21^. For further graphical examples of marker gene expression, see Supplemental Fig. 1. Queries for markers of other immune and retinal cell types, including dendritic cells, neutrophils, and T and B cells, did not identify any other major cell types in the dataset, as expected since only CD11b^+^ cells were selected for analysis.

To independently identify differences in gene expression between these clouds, we used Seurat to generate lists of abundant transcripts differentially expressed in each Cloud relative to all other cells. These lists were rife with many known marker genes for these cell types (Supplemental Table 1), including several of the markers queried in Fig. 3 and Supplemental Fig. 1. Thus, both candidate gene expression queries and unbiased transcriptional profiling support the conclusion that the four primary clouds of Fig. 3b reflect: (1) Resting Microglia, (2) Activated Microglia, (3) Monocytes, and (4) Monocyte-derived Macrophages.

### *In silico* identification of cell subtypes with different activation and proliferation states

To objectively identify transcriptionally distinct groups of cells, we used an unsupervised *in silico* graph-based clustering algorithm^22^. This algorithm identified 12 distinct clusters, corresponding to 12 putative populations (Fig. 3c). One cluster fell within the cloud deemed resting microglia (Fig. 3c, Cluster #1); four clusters fell within the cloud of activated microglia (Clusters #2–5); three clusters fell within the cloud of monocytes (Clusters #6–8); two fell within the cloud of monocyte-derived macrophages (Clusters #9–10); and two very small clusters could not be confidently identified as any immune or retinal cell type (Clusters #11–12).

**Figure 4:**
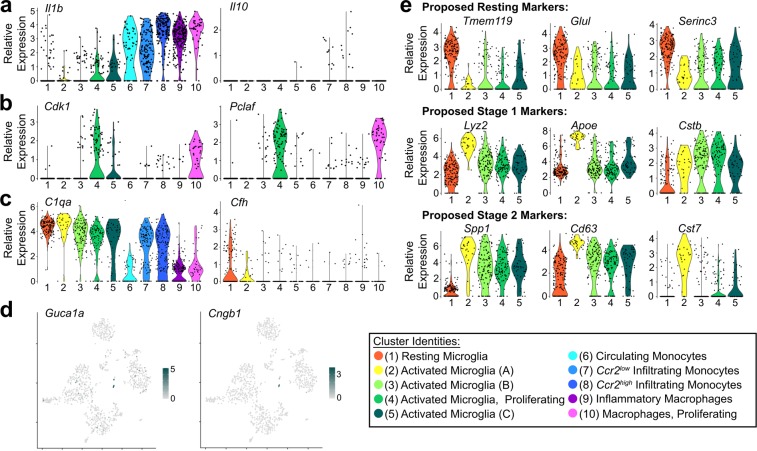
Differential gene expression between subtypes. (**a**) Representative inflammatory cytokine (*Il1b*) and anti-inflammatory cytokine (*Il10*) expression across identified clusters. (**b**) Identification of proliferative populations by expression of mitotic genes *Cdk1* and *Pclaf* (*2810417H13Rik*). (**c**) Expression of representative complement genes (*C1qa* and *Cfh*) across clusters. (**d**) Rod photoreceptor transcripts, including *Guca1a* and *Cngb1*, are found in a subset of activated microglia, particularly in several cells from Cluster #5 (activated microglia C). (**e**) Expression of disease activated (DA) microglia markers from Keren-Shaul *et al*. 2017, which suggested: *Tmem119*, *Glul*, and *Serinc3* are downregulated in DA microglia (i.e., are markers of resting microglia); *Lyz2*, *Apoe*, and *Cstb* are upregulated during Stage 1 of disease-related activation; *Spp1*, *Cd63*, and *Cst7* are upregulated in Stage 2 activation. None of the 5 microglia clusters in the present study completely align with these previously defined activation stages. For example, *Cst7* expression is low in activated microglia Clusters #3–5, despite the expression of other Stage 2 markers. Additionally, activated microglia Cluster #2 has lower expression of *Cstb* despite otherwise strong expression of several Stage 1 and Stage 2 markers. In (**a–c,e**), the color corresponds to the cluster identity and the extruded shape represents the distribution of cells with the corresponding expression levels.

To identify potential functional differences between these putative subpopulations, we used Seurat to determine the genes most differentially expressed between clusters (Supplemental Table 2 for list and *p*-values). Genes associated with specific functional states like inflammation, proliferation, and phagocytosis were enriched in particular subsets of clusters (Fig. 4). For example, expression of the inflammatory cytokines, such as *Il-1β*, was highest in monocytes and macrophages (Fig. 4a, Clusters #6–10). Cluster #6 contained cells from both control and degenerating samples that expressed markers for peripheral immune cells like *Plac8*, suggesting that these were circulating cells captured in the vasculature at the time of dissection. The majority of cells of only two of the clusters (Clusters #4, #10) expressed genes associated with cell division, such as *Cdk1* and *Pclaf*, suggesting that these subpopulations were actively proliferating (Fig. 4b). Finally, another small but distinct cluster contained some photoreceptor-specific transcripts involved in rod phototransduction, such as *Guca1a* and *Cngb1* (Fig. 4d, subset of Cluster #5). We interpret this to mean that these activated microglia had phagocytosed rods containing non-degraded mRNA immediately before tissue processing.

Microglia (Clusters #1–5) expressed many complement-related genes (Supplemental Table 2), but the relative expression patterns between clusters were not uniformly helpful in identifying potential functional subpopulations. For example, C1q component genes like *C1qa* (Fig. 4c) and some integrin subunits (data not shown) were expressed at similar levels in both control and degenerating samples, suggesting that microglia are capable of initiating the classical complement pathway in both resting and activated states. In contrast, Complement Factor H (Cfh), which inhibits the alternative complement pathway and has been linked to age-related macular degeneration^23,24^, was decreased in the degenerating sample and did not differ much between activated microglia subpopulations (Fig. 4c).

Since distinct subpopulations of activated microglia have been previously identified in Alzheimer’s disease and ALS^19^, we examined if those previously defined activated stages were sufficient to describe the subpopulations identified here (Fig. 4e). Keren-Shaul and colleagues suggested that microglia activation occurred in two stages, so we examined the expression of markers for both stages across all microglia clusters. Although many of the same resting microglia markers were expressed in our control sample (Cluster #1; Fig. 4e, top row), the relative expression profiles of activated microglia clusters did not consistently align with the activation progression observed in Alzheimer’s disease and ALS. For example, *Lyz2* and *Apoe*, two genes that were previously associated with stage one of activation in Alzheimer’s Disease, were only enriched in one of the four activated microglia clusters (Cluster #2), yet another prominent Stage 1 marker, *Cstb*, was not similarly enriched in this cluster (Fig. 4e, middle row). Clusters #3–5 expressed some genes associated with the second stage of activation, such as *Spp1* and *Cd63*, but there was a marked absence of others, such as *Cst7* (Fig. 4e, bottom row). Additionally, several of these genes and others have been identified as being differentially expressed in activated brain microglia after lysolecithin demyelination^20^. However, the relative expression and subpopulations again do not align precisely (see Supplemental Fig. 1 for further examples of lysolecithin demyelination-related genes expression during *Arr1*^*−/−*^ degeneration). These findings suggest that although some features of microglia activation may be conserved, the specific molecular phenotypes and subpopulations may depend on the tissue, type, and extent of neuronal degeneration.

### Re-examining molecular heterogeneity of the immune response in the context of degeneration

**Figure 5:**
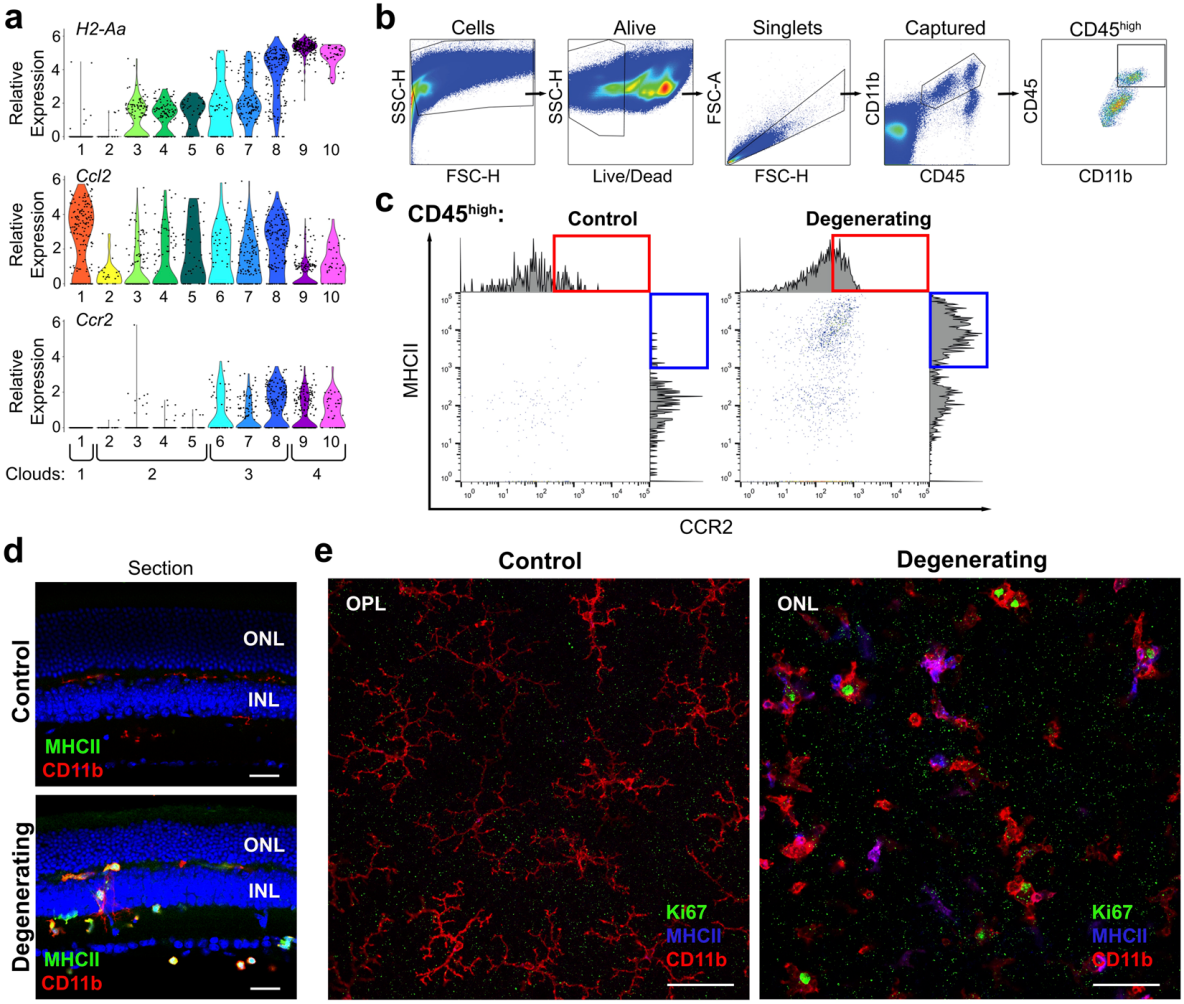
Utilizing traditional techniques in combination with scRNAseq data to understand the response of immune cell populations during acute photoreceptor degeneration. (**a**) Relative expression of *H2-Aa*, *Ccr2*, and *Ccl2* across clusters. (**b**) Complete gating strategy for identifying alive, single, captured CD11b^+^CD45^high^ cells. The gating strategy is shown here on the degenerating sample. (**c**) MHCII and CCR2 protein expression on CD11b^+^CD45^high^ cells gated for scRNAseq. There was an increase in MHCII^high^ events (blue box) and CCR2 (red box) in degenerating tissue. Histograms on the edge of plots show the distribution of events across fluorescence intensities of MHCII and CCR2. (**d**,**e**) During degeneration a subset of macrophage-like CD11b^+^ cells express MHCII. No MHCII expression is regularly detected in control *Arr1*^*−/−*^ retinas. In retinal sections, (**d**) MHCII^+^ cells can be seen in multiple retinal layers. Retinal flatmounts (**e**) clearly reveal that some, but not all, macrophage-like cells express MHCII during degenerationg, and there are no clear morphological distinctions between MHCII^+^ and MHCII^−^ macrophage-like cells. Additionally, during degeneration proliferating macrophage-like CD11b^+^ cells can be identified in the retina by nuclear Ki67 staining. Ki67^+^ nuclei are not observed in dark-reared control retinas, likely due to the very low levels of microglia turnorver at rest. Both MHCII^+^ (magenta, due to overlap of red and blue) and MHCII^−^ (red) CD11b^+^ cells with Ki67^+^ nuclei (green) can be observed in degenerating retinas. Scale bars in retinal sections indicate 25 µm; scale bars in flat mounts indicate 50 µm. ONL = outer nuclear layer; OPL = outer plexiform layer; INL = inner nuclear layer.

Translating the insights garnered from the single-cell data into the framework of retinal structure and more traditional methodologies is essential for subsequently testing and manipulating immune cell function during degeneration. As a first step, we focused on MHCII, a common marker of monocyte-derived macrophages^25^ that indeed showed prominent expression in those corresponding clusters (Fig. 5a, *H2-Aa*, Clusters #9–10). FACS detection of MHCII antibody staining revealed a dramatic increase in MHCII protein levels in CD45^+^ CD11b^+^ cells from the degenerating retina (Fig. 5c, blue box, compare control to degenerating; see Fig. 5b for gating strategy). The same MHCII antibody when applied to immunohistochemistry stained only a fraction of CD11b^+^ cells, as best illustrated by looking at a population of cells across a large retinal area in flat mounts (Fig. 5e). In retinal sections, MHCII^+^ cells were not localized in any particular layer but instead were distributed throughout the retina, from the vitreal surface to the outer plexiform layer (Fig. 5d). This suggests either that the monocyte-derived macrophages did not rapidly accumulate in the photoreceptor layers or that the differentiation from monocyte to macrophage happened well before the cells arrived at their final destination. Notably, there were no clear morphological distinctions between MHCII^+^ and MHCII^−^ populations, emphasizing the inadequacy of using morphology for discriminating between activated microglia and monocyte-derived macrophages in this instance.

Through scRNAseq we also identified two subpopulations of proliferating cells in denerating retina (Fig. 4b, Clusters #4, 10). Utilizing IHC, we confirmed the presence of mitotically active Cd11b^+^ cells using Ki67 staining in degenerating tissue. Again, Ki67^+^ cells were not localized to a particular retinal region and instead seemed equally scarce in both inner and outer retinal layers (Fig. 5e, example shown in outer retina). In contrast, Ki67^+^ nuclei were never observed in dark-reared control retinas, consistent with the scRNAseq findings.

In some cases, our scRNAseq data revealed cellular heterogeneity in the expression of genes commonly considered to be cell type-specific markers. For example, *Ccr2*, a common monocyte marker (e.g.^25^), was detectable in both monocyte and macrophage populations (Clouds #3 and #4; Fig. 5a), though indeed only statistically differentially expressed in monocyte Cloud #3 (1.5-fold higher expression; p = 1.35E-26; Supplemental Table 1). Notably,within Cloud #3 it was only differentially expressed in the inflammatory monocytes of Cluster #8 (1.8-fold higher expression, p = 3.30E-29 Supplemental Table 2), and not the other monocyte clusters. Antibody labeling and detection using FACS showed a similar modest increase in total CCR2 staining between control and degenerating retinas (Fig. 5c, red box). However, neither FACS nor scRNAseq results were as dramatic as the evident infiltration revealed by *in vivo* imaging of *Ccr2*^+/*RFP*^ retinas (Fig. 1b), possibly reflecting a difference in the lifetime of the RFP protein as compared to CCR2.

## Discussion

The present study uses scRNAseq to reveal distinct subpopulations of immune responders within the retina. In combination with *in vivo* imaging, flow cytometry, and immunohistochemistry, our results show that microglial activation, monocyte infiltration, and monocyte differentiation into macrophages can occur concurrently and rapidly, within 48 hours after the onset of photoreceptor degeneration. Here we describe the distinguishing molecular features of these subpopulations and discuss the consequences of these findings for retinal degeneration and neuroinflammation more generally.

### Distinguishing hallmarks of immune subpopulations in the degenerating retina

**Figure 6:**
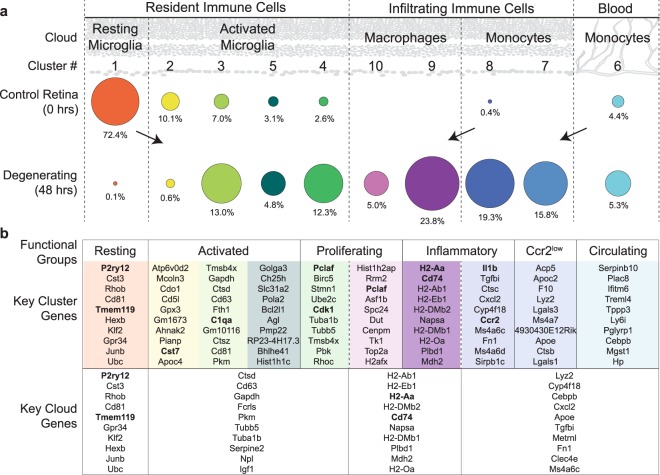
Summary of immune subpopulations identified by scRNAseq. (**a**) Comparison of the relative population sizes organized by cloud and cluster number for control (0 hours of light exposure) and degenerating (48 hours of light exposure) tissue. In control retina, almost all of the immune cells were resident microglia, with resting microglia being the dominant type. In denerating tissue, most immune cells were infiltrating monocytes and monocyte-derived macrophages, while only a third were resident microglia, demonstrating that in the degenerating retina, microglia activation occurs concurrently with monocyte infiltration and differentiation into macrophages. The size of each dot corresponds to to the number of cells captured; the percent of cells within each condition is given. (**b**) Groups based on gene expression profiles for each cluster. The top 10 significantly differentialy expressed genes for each cluster and cloud are given (sorted by adjusted p-value, see Supplemental Tables 1 and 2 for an extended list, see Methods for description of how lists were created). Expression of genes in bold are also shown graphically throughout Figs 3–5.

Rigorous analysis of the scRNAseq dataset generated here revealed the presence of distinct populations and subpopulations of both invading and resident myeloid cells, consistent with *in vivo* imaging (Fig. 1b), FACS analysis (Figs 2 and 5b,c), and immunohistochemistry (Figs 1a and 5d,e). Figure 6a outlines the distribution of immune subpopulations by location (retina vs. blood), condition (control vs degenerating), cloud group, and cluster number. In control retina, almost all of the immune cells were resident microglia; the majority (72.4%) of which were resting, compared to 22.8% activated. The remaining immune cells in control retina were circulating monocytes trapped in the blood stream (4.4%). Remarkably, in degenerating tissue the majority of immune cells (64%) were peripheral in origin, and at the time point investigated here were comprised roughly equally of infiltrating monocytes (35.1%) and monocyte-derived macrophages (28.8%). Only 30.7% of the immune cells in the degenerating retina were activated, resident microglia.

Statistical comparison of transcriptional profiles revealed distinct subpopulations with clear and often dramatic differences in gene expression between them. Figure 6b lists the top 10 significantly differentially expressed genes for each cluster and cloud, sorted by the adjusted p-value (see Supplemental Tables 1 and 2 for an extended list and all p-values) and categorized into functional groups based on gene expression in the cluster subpopulations. For example, proliferating microglia and monocytes (Clusters #4 and #10, respectively) both express significantly higher levels of *Pclaf* (*2810417H13Rik*) compared to all other cells, indicating that these cells were undergoing DNA replication and establishing their status in the proliferating functional group. Nonetheless, the molecular profile of numerous other genes within these clusters allowed us to simultaneously classify them into two distinct subpopulations of activated microglia and macrophages. Similarly, the molecular profile of Clusters #1–5 clearly identify these as Resting (#1) and Activated (#2–5) microglia; however the more subtle differences between Clusters #2, #3, and #5 raise the question as to whether the activated microglia clusters represent stages of one continuous activation pathway or functionally distinct pathways. Finally, the key cloud genes (Fig. 6b, bottom) emphasize commonalities in gene expression across the broader cell groups: Resting Microglia, Activated Microglia, Monocytes, and Monocyte-derived Macrophages and are derived from Supplemental Table 1.

### Understanding molecular heterogeneity is critical for potential functional analysis

In addition to revealing subpopulations of each cell class, the scRNAseq analysis allows inference about the potential function(s) of individual subpopulations to ascertain the role they play during neurodegeneration. For example, Macrophages (Cloud #4, Clusters #9, #10) showed significantly higher levels of *H2-Aa* expression, an MHCII gene associated with antigen processing and presentation compared to all other groups (Fig. 5a, Supplemental Tables 1, 2). Retinal MHCII^+^ cells, especially in the subretinal space, have been described in models of RPE death^6^, and after light injury in *Cx3cr1*^*−/− *^^7^, *RPE65*^*Leu/Leu*^, and wild-type mice^8^. Our immunohistochemistry revealed that MHCII^+^ cells were localized throughout all of the retinal layers and were not morphologically distinct from other CD11b^+^ cells. The differences in the localization and appearance of MHCII^+^ cells in these various models of degeneration may reflect differences in the site of infiltration, either across Bruch’s membrane and RPE, or, as in the present study, across retinal vessels.

Another potential functional inference that can be drawn from the data is the role of CCL2-CCR2 signaling, which is widely known to recruit systemic immune cells during retinal degeneration. Here we found that *Ccl2* was detectable in all of the immune subgroups (Fig. 5a), and notably not differentially increased in activated microglia, suggesting they are not the source of CCL2 to recruit infiltrating monocytes at this time point. Conversely, Monocytes (Cloud #3) and Macrophages (Cloud #4) showed expression of *Ccr2*, the CCL2 receptor (Fig. 5a), with considerable variability in expression between Clusters #6–10. This variability may reflect the rapid degradation of CCR2 and downregulation of *Ccr2* expression after extravasation into the retinal parenchyma. Indeed, in the *Cx3cr1*^*−/−*^ model of age-related macular degeneration, it has been shown that monocytes downregulate *Ccr2* rapidly when differentiating into macrophages^7^.

Surprisingly, we found a distinct lack of anti-inflammatory markers, such as *Il10* (Fig. 4a), in all cells, even in the resting microglia population. Because monocytes and macrophages are capable of adopting both pro- and anti-inflammatory phenotypes^25^, it is possible that anti-inflammatory cells may play a role in the response to photoreceptor degeneration at later time points, or as the rate of degeneration slows and the tissue enters a homeostatic “recovery” phase. It is also possible that anti-inflammatory signaling is more prevalent in slower models of retina degeneration.

### Implications for photoreceptor degeneration and other retinal degenerative diseases

Many mouse models have been described with genetic and/or phenotypic similarities to human retinal degenerative diseases with varying ages of onset and speed of degeneration^26–28^. While all models involve activation of resident microglia, the potential involvement of the peripheral immune system remains unclear. In some instances, such as in cases of RPE and/or light damage, monocytes and macrophages have been detected in the degenerating retina^5,8^, though it has not been resolved whether they enter through the retinal or choroidal vasculature.

Arrestin-1 is distinctly a photoreceptor-specific protein that is not expressed in leukocytes^29,30^ or other cells of the retina^31^. *Arr1* binds photoexcited rhodopsin^32^, quenching its ability to activate G-protein in the phototransduction cascade of vertebrate rods. Mice lacking *Arr1* have normal retinal morphology when reared in complete darkness, yet when they are exposed to light, unrestrained rhodopsin signaling causes photoreceptors to rapidly degenerate over the course of a few days^12,15,17^ even in relatively dim light^18^. Thus, the *Arr1*^*−/−*^ mouse serves as a model for time-locked, light-induced photoreceptor degeneration, allowing the acute response of both resident and infiltrating immune cells to be followed consistently in time. The ability to chart the immune response to a time-locked degeneration onset is especially critical for studying the differentiation of monocytes into macrophages within the CNS, and how these interactions may change over time. ScRNAseq in time-locked models will be important to determine how microglia and infiltrating immune cells re-establish surveillance in degenerated retina and their immune responses to therapies and subsequent insults related to aging.

In summary, scRNAseq revealed a previously immeasurable degree of heterogeneity in the myeloid response to cell-autologous degeneration of neurons within the central nervous system, including changes in the local resident populations and an invasion of immune cells from the periphery (Fig. 6). The complex differences between populations observed here highlight the necessity of utilizing unbiased high-throughput and high-dimensional molecular techniques like scRNAseq in combination with techniques like immunohistochemistry, *in vivo* imaging or ultimately spatial transcriptomics^33,34^.

## Methods

### Animals

Mice were cared for and handled in accordance with the National Institutes of Health guidelines for the care and use of experimental animals and under approved protocols by the UC Davis Institutional Animal Care and Use Committee. *Arr1*^*−/−*^ mice^12,14^ were born and maintained in constant darkness before exposure to light (200 lux, 48 hours). Homozygous *Cx3cr1*^*GFP/GFP*^ (strain 005582)^35^ and *Ccr2*^*RFP/RFP*^ (strain 017586)^36^ knock-in mice were obtained from The Jackson Laboratory, and crossed with the *Arr1*^*−/−*^ to obtain fluorescent knock-in heterozygotes in a homozygous *Arr1*^*−/−*^ background (*Arr1*^*−/−*^*Cx3cr1*^*+/GFP*^ and *Arr1*^*−/−*^*Ccr2*^*+/RFP*^, respectively). All mice used in this study were between 2–4 months old. Although there are reports of sex differences in microglia function and reactivity^37^, no differences were observed in immunohistochemistry or *in vivo* microglial imaging experiments (data not shown); thus, both male and female animals were used and the results combined. To avoid sex-specific differences in gene expression, only retinas from female mice were used for scRNAseq. Six littermates (3 dark-reared controls and 3 degenerating, 48 hours light-exposed mice) were used for single-cell sequencing in order to capture a sufficient number of high quality cells, ideally approximately 200 cells of any significant immune cell type^38^. Female littermates were randomly divided between control and degenerating groups.

### Immunohistochemistry

Immunohistochemistry was performed as previously described^39^. Briefly, mice were sacrificed by carbon dioxide euthanasia and eyes enucleated and submerged in 4% paraformaldehyde at room temperature. After 5 minutes of fixation, the cornea and lens were removed, and the eyecups were fixed for an additional 20–25 minutes. For retinal sections, fixed eyecups were embedded in agarose and sectioned on a vibratome (Leica) at a thickness of 150 µm. Sections were blocked at room temperature in normal serum, incubated in primary antibody solution at 4 °C overnight, washed in PBS 3 times for 15 minutes each, and incubated in secondary antibody solution at room temperature for 1.5–2 hours before an additional 3 PBS washes and mounting with ProLong Diamond Antifade (Invitrogen). For retinal flatmounts, retinas were removed from fixed eyecups, followed by incubation in 1% Triton X-100 in PBS overnight at 4 °C, and then blocked with normal serum for 2 hours at 37 °C. Retinas were incubated in primary antibody solution overnight at 4 °C, washed 3 times in PBT for 15–30 minutes at room temperature, and incubated in secondary antibody solution for 1.5–2 hours at 37 °C before 3 PBT washes and mounting with ProLong Diamong Antifade with DAPI (Invitrogen). Tissue was stained for Ki67 using an antibody raised in rabbit (1:300; Abcam), followed by Alexa Fluor-conjugated secondary antibody (1:300; Invitrogen); rat antibodies against CD11b and MHCII (1:300 and 1:200 respectively; BioLegend) were preconjugated to Alexa Fluors and added to the seconday antibody solution; DAPI (Invitrogen) was also added to the secondary antibody solution (2 drops/mL). All sections and flatmounts were imaged using a Nikon A1 confocal microscope.

### *In vivo* imaging with scanning laser ophthalmoscopy

A custom-built scanning laser ophthalmoscopy (SLO) system was used to image GFP^+^ and RFP^+^ cells within the retina, simultaneously collecting the reflectance and fluorescence images^40^. For imaging, mice were anesthetized with 2–2.5% isoflurane and positioned on a heating pad (37 °C) with a micropositioner (Bioptigen, Morrisville, NC) that allowed rotational and translational adjustment for positioning the mouse with respect to the contact lens. The pupils were dilated and cyclopleged with tropicamide and phenylephrine, and the corneal surface wetted with Gel Tears hypromellose gel (GenTeal Tears Severe, Alcon). Gel Tears helped maintain a homogeneous refractive surface between the cornea and the custom 0 diopter contact lens (Unicon Corporation, Osaka, Japan). GFP and RFP excitation was achieved with 488 nm and 561 nm lasers, respectively. Images were collected over 51° of visual angle at 43 µm per degree. In Fiji^41^, images were registered^42^, averaged, background subtracted, and pseudo-colored for presentation.

### Fluorescence activated cell sorting (FACS)

FACS was performed using a protocol modified from an established method^8,17^. After dissection, each retina was incubated in 1 mL of digestion buffer containing: Hank’s Balanced Salt Solution (10–547 F, Lonza), 5% Fetal Bovine Serum (FBS; 35010CV, Corning), 10 mM HEPES, 0.7 mg/mL calcium chloride, 1.5 mg/mL Collagenase A (10103586001, Roche), and 0.1 mg/mL DNase I (10104159001, Roche) at 37 °C for 15 minutes. Following incubation, each retina was gently dissociated and the resulting single-cell suspension was washed, filtered through a 70 µm cell strainer, centrifuged at 350xg for 5 minutes, and resuspended in PBS. Cells were stained for viability (Zombie Viability NIR, Biolegend), and blocked with Fc light chain antibodies (eBiosciences), supplemented with normal rat serum and normal mouse serum. Cells were then incubated with anti-mouse CD11b conjugated to BV605, anti-mouse CD45 conjugated to PE, anti-mouse CCR2 conjugated to PE/Cy7, and anti-mouse AI/IE (MHCII) conjugated to BV711 (all Biolegend). Cell suspensions were washed in PBS containing 0.5% Bovine Serum Albumin (BSA), centrifuged at 350 × g for 5 minutes, and resuspended in 0.5% BSA in PBS with 1:50 EDTA. All retinas were processed separately, until being combined by group (6 retinas from 3 dark-reared, control mice and 6 retinas from 3 light-exposed, degenerating mice) immediately before sorting. Cells were sorted into 40 µL of ice cold DMEM supplemented with 10% FBS using the MoFlo Astrios cell sorter (Beckman) at the UC Davis Flow Cytometry Core, gating for alive CD11b^+^CD45^+^ cells. Further analysis was performed using FlowJo.

### Single-cell RNA library preparation and sequencing

Sequencing libraries were prepared from the sorted CD11b^+^CD45^+^ cells from each group (control and degenerating samples) using the 10X Genomics system (Chromium) at the UC Davis DNA Technologies and Expression Core according to manufacturer recommendations. The two resulting cDNA libraries were sequenced on a NextSeq (Illumina) system running 150 cycles of paired-end reads at the UC Davis DNA Technologies and Expression Core according to manufacturer recommendations. The transcript read lengths were 98 bp, and the sample index, cell barcode, and UMI read lengths were 8 bp, 14 bp, and 10 bp respectively. Cells were sequenced at a mean depth of 474,191 reads per cell in the control library and 225,889 reads per cell in the degenerating library. The scRNAseq dataset generated and analyzed here is available in the NCBI Gene Expression Omnibus (GEO) repository, accession number GSE121081.

### scRNAseq Analysis

Initial sequence processing, including barcode processing, transcript alignment, and generation of gene-barcode matrices were performed using Cell Ranger (Chromium) at the UC Davis Bioinformatics Core. Further processing and analysis was performed in R, primarily with the Seurat package unless otherwise specified^43–45^. Cells were examined for quality control, removing those with unique gene counts over 3,000 (to remove potential doublets from the dataset) and under 500 (to remove spurious contamination). Data were then normalized for unique molecular identifier (UMI) counts per cell and mitochondrial genes following the standard Seurat workflow. After principal components analysis, the transcript expression from the top ten principal components was used to perform *t*-distributed stochastic neighbor embedding (tSNE) dimensional reduction. Clusters were identified using a graph-based local moving algorithm with a resolution of 1.5^22^. This resolution was selected after trying a wide range of resolution values (0.6–1.6) and decided upon in consultation with the UC Davis Bioinformatics Core, based upon the generation of a similar number of clusters across several resolutions and reasonable numbers of differentially expressed genes between clusters. Expression of marker genes were identified using the FindMarkers function in Seurat. This function identifies genes differentially expressed in a given group (either a cluster or cloud) compared to all other cells in the dataset using a non-parametric Wilcoxon rank sum test followed by Bonferroni correction. For each cluster or cloud, p-value adjustments were based on the total number of genes expressed in at least 25% of the cells in the cluster or cloud being tested.

## Supplementary information

Supplement

## References

[1] Herz J, Filiano AJ, Smith A, Yogev N, Kipnis J (2017). Myeloid Cells in the Central Nervous System. Immunity.

[2] Wolf SA, Boddeke HW, Kettenmann H (2017). Microglia in Physiology and Disease. Annu Rev Physiol.

[3] Ousman SS, Kubes P (2012). Immune surveillance in the central nervous system. Nat Neurosci.

[4] Baufeld C, O’Loughlin E, Calcagno N, Madore C, Butovsky O (2018). Differential contribution of microglia and monocytes in neurodegenerative diseases. J Neural Transm (Vienna).

[5] Guillonneau X (2017). On phagocytes and macular degeneration. Prog Retin Eye Res.

[6] Ma W (2017). Monocyte infiltration and proliferation reestablish myeloid cell homeostasis in the mouse retina following retinal pigment epithelial cell injury. Sci Rep.

[7] Sennlaub F (2013). CCR2(+) monocytes infiltrate atrophic lesions in age-related macular disease and mediate photoreceptor degeneration in experimental subretinal inflammation in Cx3cr1 deficient mice. EMBO Mol Med.

[8] O’Koren EG, Mathew R, Saban DR (2016). Fate mapping reveals that microglia and recruited monocyte-derived macrophages are definitively distinguishable by phenotype in the retina. Sci Rep.

[9] Reyes NJ, O’Koren EG, Saban DR (2017). New insights into mononuclear phagocyte biology from the visual system. Nat Rev Immunol.

[10] McMenamin, P. G., Saban, D. & Dando, S. J. Immune cells in the retina and choroid: Two different tissue environments that require different defenses and surveillance. *Prog Retin Eye Res*, 10.1016/j.preteyeres.2018.12.002 (2018). 10.1016/j.preteyeres.2018.12.002 PMC7321801 30552975

[11] Hickman SE (2013). The microglial sensome revealed by direct RNA sequencing. Nat Neurosci.

[12] Xu J (1997). Prolonged photoresponses in transgenic mouse rods lacking arrestin. Nature.

[13] Wilden U, Kuhn H (1982). Light-dependent phosphorylation of rhodopsin: number of phosphorylation sites. Biochemistry.

[14] Chen J, Simon MI, Matthes MT, Yasumura D, LaVail MM (1999). Increased susceptibility to light damage in an arrestin knockout mouse model of Oguchi disease (stationary night blindness). Invest Ophthalmol Vis Sci.

[15] Hao W (2002). Evidence for two apoptotic pathways in light-induced retinal degeneration. Nat Genet.

[16] Wang T, Chen J (2014). Induction of the unfolded protein response by constitutive G-protein signaling in rod photoreceptor cells. J Biol Chem.

[17] Karlen, S. J. Miller, E. B., Wang, X., Levine, E. S., Zawadzki, R. J. & Burns, M.E. Monocyte infiltration rather than microglia proliferation dominates the early immune response to rapid photoreceptor degeneration. *Journal of Neuroinflammation* **15,** 344, 10.1186/s12974-018-1365-4 (2018). 10.1186/s12974-018-1365-4 PMC7659426 30553275

[18] Levine ES (2014). Rapid light-induced activation of retinal microglia in mice lacking Arrestin-1. Vision Res.

[19] Keren-Shaul H (2017). A Unique Microglia Type Associated with Restricting Development of Alzheimer’s Disease. Cell.

[20] Hammond, T. R. *et al*. Single-Cell RNA Sequencing of Microglia throughout the Mouse Lifespan and in the Injured Brain Reveals Complex Cell-State Changes. *Immunity*, 10.1016/j.immuni.2018.11.004 (2018). 10.1016/j.immuni.2018.11.004 PMC6655561 30471926

[21] Yu YR (2016). A Protocol for the Comprehensive Flow Cytometric Analysis of Immune Cells in Normal and Inflamed Murine Non-Lymphoid Tissues. PLoS One.

[22] Macosko EZ (2015). Highly Parallel Genome-wide Expression Profiling of Individual Cells Using Nanoliter Droplets. Cell.

[23] McHarg S, Clark SJ, Day AJ, Bishop PN (2015). Age-related macular degeneration and the role of the complement system. Mol Immunol.

[24] Calippe B (2017). Complement Factor H Inhibits CD47-Mediated Resolution of Inflammation. Immunity.

[25] Yang J, Zhang L, Yu C, Yang XF, Wang H (2014). Monocyte and macrophage differentiation: circulation inflammatory monocyte as biomarker for inflammatory diseases. Biomark Res.

[26] Kiser PD, Palczewski K (2016). Retinoids and Retinal Diseases. Annu Rev Vis Sci.

[27] Chang B (2002). Retinal degeneration mutants in the mouse. Vision Res.

[28] Levy O (2015). APOE Isoforms Control Pathogenic Subretinal Inflammation in Age-Related Macular Degeneration. J Neurosci.

[29] Shay T, Kang J (2013). Immunological Genome Project and systems immunology. Trends Immunol.

[30] Heng TS, Painter MW (2008). & Immunological Genome Project, C. The Immunological Genome Project: networks of gene expression in immune cells. Nat Immunol.

[31] Siegert S (2012). Transcriptional code and disease map for adult retinal cell types. Nat Neurosci.

[32] Wilden U, Hall SW, Kuhn H (1986). Phosphodiesterase activation by photoexcited rhodopsin is quenched when rhodopsin is phosphorylated and binds the intrinsic 48-kDa protein of rod outer segments. Proc Natl Acad Sci USA.

[33] Stahl PL (2016). Visualization and analysis of gene expression in tissue sections by spatial transcriptomics. Science.

[34] Strell, C. *et al*. Placing RNA in context and space - methods for spatially resolved transcriptomics. *FEBS J*, 10.1111/febs.14435 (2018). 10.1111/febs.14435 29542254

[35] Jung S (2000). Analysis of fractalkine receptor CX(3)CR1 function by targeted deletion and green fluorescent protein reporter gene insertion. Mol Cell Biol.

[36] Saederup N (2010). Selective chemokine receptor usage by central nervous system myeloid cells in CCR2-red fluorescent protein knock-in mice. PLoS One.

[37] Lenz KM, McCarthy MM (2015). A starring role for microglia in brain sex differences. Neuroscientist.

[38] Ziegenhain C (2017). Comparative Analysis of Single-Cell RNA Sequencing Methods. Mol Cell.

[39] Ronning KE (2018). Loss of cone function without degeneration in a novel Gnat2 knock-out mouse. Exp Eye Res.

[40] Zhang P (2015). *In vivo* wide-field multispectral scanning laser ophthalmoscopy-optical coherence tomography mouse retinal imager: longitudinal imaging of ganglion cells, microglia, and Muller glia, and mapping of the mouse retinal and choroidal vasculature. J Biomed Opt.

[41] Schindelin J (2012). Fiji: an open-source platform for biological-image analysis. Nat Methods.

[42] Thevenaz P, Ruttimann UE, Unser M (1998). A pyramid approach to subpixel registration based on intensity. IEEE Trans Image Process.

[43] R: A language and environment for statistical computing (R Foundation for Statistical Computing, Vienna, Austria, 2008).

[44] Satija R, Farrell JA, Gennert D, Schier AF, Regev A (2015). Spatial reconstruction of single-cell gene expression data. Nat Biotechnol.

[45] Butler A, Hoffman P, Smibert P, Papalexi E, Satija R (2018). Integrating single-cell transcriptomic data across different conditions, technologies, and species. Nat Biotechnol.

