# Supplementary material for: Molecular profiling of resident and infiltrating mononuclear phagocytes during rapid adult retinal degeneration using single-cell RNA sequencing
Source: Sci Rep. 2019 Mar 19;9:4858. doi: 10.1038/s41598-019-41141-0 (PMC6425014; doi:10.1038/s41598-019-41141-0)
Supplement: Supplementary file 1 — Supplement [file 41598_2019_41141_MOESM1_ESM.docx]

Supplemental Information:

**Molecular profiling of resident and infiltrating mononuclear phagocytes during rapid adult retinal degeneration using single-cell RNA sequencing**

Kaitryn E. Ronning^1^, Sarah J. Karlen^2^, Eric B. Miller^1^, and Marie E. Burns^1,2,3^ *

^1^Center for Neuroscience, ^2^Dept. of Cell Biology and Human Anatomy, and ^3^Dept. Ophthalmology & Vision Science; University of California Davis; Davis CA 95616.

*Corresponding author, contact at meburns@ucdavis.edu

Contents:

Supplemental Figure 1

Supplemental Table 1

Supplemental Table 2

Supplemental Figure 1.

**Supplemental Figure 1.** Many commonly used marker genes of monocytes and macrophage-like cells are expressed in immune cells before and/or during retinal degeneration. Relative expression is indicated on tSNE plots, with the highest expression in saturated color and the lowest expression in gray.

**Supplemental Table 1.** Top 35 differentially expressed genes for cells in each cloud (defined in Figure 3B), listed in ascending order based on adjusted p-value. Differentially expressed genes were identified using the FindMarkers function in Seurat, using a Wilcoxon rank sum test followed by Bonferroni correction. For each cloud, all cells within that cloud were compared to all other cells outside of the cloud. The log_2_FC expression indicates the log_2_ fold-change of the average increase in expression in cells in that cloud compared to the average expression across all other cells. Genes were only considered if they were expressed in at least 25% of the cells within that cloud. Also shown are the percent of cells within and outside of the cloud that express a given gene.

1. Cloud 1

| **Gene symbol** | **Log_2_FC expression** | **Adjusted p-value** | **Cells expressing gene in Cloud (%)** | **All other cells expressing (%)** |
| --- | --- | --- | --- | --- |
| P2ry12 | 2.776 | 4.15E-135 | 98.1% | 13.6% |
| Cst3 | 2.289 | 2.54E-82 | 100.0% | 97.7% |
| Rhob | 2.031 | 4.33E-80 | 92.0% | 26.3% |
| Cd81 | 1.976 | 1.18E-78 | 100.0% | 64.3% |
| Tmem119 | 2.012 | 5.45E-78 | 85.8% | 21.4% |
| Gpr34 | 1.902 | 7.88E-78 | 77.2% | 13.0% |
| Klf2 | 2.412 | 2.42E-77 | 99.4% | 46.4% |
| Hexb | 1.675 | 1.23E-76 | 100.0% | 90.3% |
| Junb | 1.774 | 4.02E-76 | 100.0% | 88.3% |
| Ubc | 1.647 | 1.87E-72 | 99.4% | 89.1% |
| Siglech | 1.521 | 1.36E-68 | 50.6% | 4.0% |
| Jun | 2.394 | 2.13E-67 | 98.1% | 66.4% |
| Fos | 2.073 | 5.42E-67 | 99.4% | 79.7% |
| Sparc | 1.552 | 4.72E-66 | 78.4% | 16.2% |
| Jund | 1.673 | 1.39E-65 | 98.8% | 75.8% |
| Egr1 | 1.850 | 1.44E-65 | 94.4% | 39.8% |
| Adrb2 | 2.309 | 8.66E-64 | 72.8% | 16.1% |
| Sepp1 | 1.764 | 6.34E-59 | 93.8% | 42.8% |
| Ctss | 1.036 | 2.27E-57 | 100.0% | 96.7% |
| Ccl3 | 2.187 | 3.26E-57 | 95.7% | 67.6% |
| Selplg | 1.787 | 6.87E-56 | 92.6% | 52.3% |
| Ltc4s | 1.341 | 7.61E-56 | 61.1% | 10.3% |
| C1qc | 1.213 | 1.16E-55 | 100.0% | 79.2% |
| Il1a | 1.803 | 1.69E-55 | 77.2% | 21.9% |
| Dusp1 | 1.498 | 1.43E-53 | 99.4% | 81.7% |
| Ier2 | 1.486 | 1.01E-48 | 98.8% | 74.0% |
| C1qb | 1.089 | 1.72E-48 | 100.0% | 84.4% |
| Ccl4 | 2.036 | 2.89E-48 | 96.3% | 73.9% |
| Glul | 1.608 | 1.95E-47 | 86.4% | 36.9% |
| Serinc3 | 1.237 | 4.81E-46 | 95.1% | 61.5% |
| Serpine2 | 1.317 | 5.92E-46 | 85.8% | 33.1% |
| Fosb | 1.506 | 1.10E-45 | 81.5% | 30.5% |
| Trem2 | 1.286 | 2.33E-45 | 94.4% | 56.5% |
| C1qa | 1.051 | 3.83E-45 | 100.0% | 77.2% |
| Btg2 | 1.756 | 7.27E-43 | 87.7% | 40.2% |

1. Cloud 2 – Activated microglia

| **Gene Symbol** | **Log_2_FC expression** | **Adjusted p-value** | **Cells expressing gene in Cloud (%)** | **All other cells expressing (%)** |
| --- | --- | --- | --- | --- |
| Ctsd | 1.500 | 1.15E-52 | 94.4% | 88.1% |
| Cd63 | 1.369 | 2.14E-51 | 86.7% | 54.8% |
| Gapdh | 0.839 | 6.29E-39 | 92.6% | 80.1% |
| Fcrls | 1.332 | 7.04E-37 | 59.3% | 23.8% |
| Pkm | 0.987 | 1.80E-34 | 89.2% | 76.8% |
| Tubb5 | 1.118 | 2.21E-33 | 79.6% | 52.4% |
| Tuba1b | 0.948 | 1.79E-31 | 82.7% | 60.8% |
| Serpine2 | 0.896 | 3.20E-31 | 67.0% | 30.0% |
| Npl | 1.041 | 5.91E-30 | 35.8% | 7.6% |
| Igf1 | 1.092 | 2.28E-29 | 31.2% | 5.4% |
| Cd81 | 0.368 | 2.42E-28 | 90.1% | 61.1% |
| Fth1 | 0.765 | 6.79E-28 | 100.0% | 99.6% |
| Stmn1 | 1.262 | 8.42E-27 | 49.7% | 19.8% |
| Aldoa | 0.874 | 2.80E-26 | 88.0% | 82.5% |
| C1qa | 0.677 | 4.44E-25 | 92.3% | 75.8% |
| Rhoc | 1.079 | 1.33E-24 | 40.7% | 13.0% |
| Birc5 | 1.077 | 2.07E-24 | 28.4% | 5.2% |
| C1qc | 0.624 | 8.46E-24 | 92.9% | 77.9% |
| Cst7 | 1.300 | 9.56E-24 | 33.3% | 8.6% |
| Tmsb4x | 0.692 | 6.36E-22 | 99.4% | 100.0% |
| Syngr1 | 1.122 | 1.85E-21 | 60.2% | 33.0% |
| Ctsz | 0.662 | 1.15E-20 | 93.2% | 94.6% |
| Mt1 | 1.345 | 2.32E-20 | 66.7% | 47.4% |
| Prdx1 | 0.618 | 2.84E-20 | 91.0% | 81.1% |
| Ctsb | 0.689 | 1.18E-19 | 96.3% | 96.9% |
| Gm10116 | 0.507 | 7.12E-18 | 99.1% | 99.9% |
| C1qb | 0.481 | 1.09E-17 | 96.6% | 82.6% |
| Mif | 0.915 | 1.48E-17 | 72.5% | 54.1% |
| Ftl1 | 0.477 | 1.63E-17 | 99.7% | 100.0% |
| Timp2 | 1.251 | 1.00E-16 | 57.1% | 33.5% |
| 2810417H13Rik | 0.872 | 2.25E-16 | 30.9% | 9.4% |
| Lpl | 1.266 | 7.77E-16 | 35.5% | 13.8% |
| Cd9 | 0.620 | 1.55E-15 | 88.6% | 81.4% |
| Hmgn1 | 1.253 | 2.28E-15 | 51.9% | 30.2% |
| Ctsl | 0.854 | 3.55E-15 | 59.0% | 36.6% |

1. Cloud 3 - Monocytes

| **Gene symbol** | **Log_2_FC expression** | **Adjusted p-value** | **Cells expressing gene in Cloud (%)** | **All other cells expressing (%)** |
| --- | --- | --- | --- | --- |
| Lyz2 | 1.828 | 6.44E-96 | 100.0% | 94.2% |
| Cyp4f18 | 1.378 | 2.79E-73 | 76.1% | 24.6% |
| Cebpb | 1.491 | 8.71E-71 | 97.7% | 72.2% |
| Cxcl2 | 1.641 | 1.21E-68 | 92.9% | 59.7% |
| Apoe | 1.019 | 1.90E-67 | 100.0% | 99.0% |
| Tgfbi | 1.119 | 7.10E-60 | 80.4% | 32.0% |
| Metrnl | 1.324 | 1.83E-58 | 63.9% | 16.5% |
| Fn1 | 1.130 | 1.87E-54 | 56.0% | 12.4% |
| Clec4e | 1.168 | 4.51E-49 | 42.9% | 6.3% |
| Ms4a6c | 0.930 | 1.48E-46 | 86.1% | 43.7% |
| Chil3 | 1.489 | 8.77E-44 | 33.2% | 3.0% |
| Lgals1 | 0.895 | 1.34E-43 | 84.9% | 50.5% |
| Acp5 | 1.404 | 1.37E-43 | 59.7% | 20.6% |
| Lst1 | 0.872 | 2.71E-43 | 54.0% | 13.5% |
| Fcer1g | 0.575 | 1.45E-42 | 99.7% | 92.1% |
| Cyba | 0.659 | 9.08E-42 | 98.3% | 86.7% |
| Pla2g7 | 0.926 | 1.39E-40 | 39.5% | 6.5% |
| Msrb1 | 0.982 | 3.76E-39 | 70.2% | 30.2% |
| Prdx5 | 0.816 | 5.93E-39 | 92.0% | 66.6% |
| Plaur | 0.983 | 3.54E-37 | 72.4% | 32.8% |
| Ifi27l2a | 1.106 | 4.16E-35 | 71.9% | 33.4% |
| Il1b | 0.865 | 4.07E-34 | 88.4% | 51.2% |
| Lgals3 | 0.863 | 9.74E-34 | 92.3% | 63.7% |
| Ms4a7 | 1.039 | 1.03E-33 | 48.6% | 14.2% |
| 4930430E12Rik | 0.509 | 5.38E-31 | 34.7% | 6.5% |
| Sirpb1c | 0.808 | 1.37E-30 | 39.2% | 9.2% |
| Gm10116 | 0.456 | 5.00E-30 | 100.0% | 99.5% |
| Ifitm3 | 0.899 | 1.86E-28 | 88.4% | 51.4% |
| Arg1 | 1.450 | 3.25E-28 | 31.5% | 6.2% |
| Ccrl2 | 0.771 | 1.32E-27 | 71.6% | 37.3% |
| Psap | 0.650 | 6.18E-27 | 98.9% | 91.7% |
| Ccr2 | 0.628 | 1.35E-26 | 53.1% | 20.1% |
| Thbs1 | 0.650 | 2.11E-26 | 26.1% | 3.6% |
| Fcgr2b | 0.604 | 6.04E-26 | 60.5% | 25.8% |
| Sdcbp | 0.562 | 5.10E-25 | 94.0% | 78.9% |

1. Cloud 4 - Macrophages

| **Gene symbol** | **Log_2_FC expression** | **Adjusted p-value** | **Cells expressing gene in Cloud (%)** | **All other cells expressing (%)** |
| --- | --- | --- | --- | --- |
| H2-Ab1 | 2.156 | 1.27E-111 | 100.0% | 71.5% |
| H2-Eb1 | 2.170 | 2.00E-111 | 100.0% | 56.2% |
| H2-Aa | 2.101 | 3.80E-111 | 100.0% | 62.2% |
| H2-DMb2 | 2.000 | 4.27E-111 | 88.3% | 20.6% |
| Cd74 | 1.984 | 1.55E-108 | 100.0% | 80.4% |
| Napsa | 1.744 | 2.49E-99 | 89.1% | 23.5% |
| H2-DMb1 | 1.478 | 1.27E-88 | 98.4% | 45.0% |
| Plbd1 | 1.378 | 3.92E-81 | 92.6% | 30.4% |
| Mdh2 | 1.560 | 4.93E-80 | 96.9% | 57.6% |
| H2-Oa | 1.606 | 2.11E-77 | 54.3% | 5.0% |
| Cfp | 1.463 | 5.48E-77 | 85.2% | 27.2% |
| H2-DMa | 1.214 | 1.03E-69 | 98.4% | 56.8% |
| Il1r2 | 1.410 | 9.93E-69 | 59.4% | 9.8% |
| Crip1 | 1.139 | 1.47E-66 | 97.3% | 54.5% |
| Itgb7 | 1.096 | 3.57E-65 | 50.8% | 5.5% |
| Lsp1 | 0.968 | 5.58E-60 | 88.3% | 35.1% |
| Bhlhe40 | 1.172 | 5.64E-60 | 80.9% | 28.9% |
| Tmsb10 | 0.911 | 1.65E-58 | 99.2% | 76.7% |
| Epcam | 1.092 | 4.64E-58 | 35.5% | 1.5% |
| Qpct | 1.566 | 2.44E-57 | 61.3% | 14.6% |
| S100a11 | 1.255 | 1.16E-56 | 77.7% | 25.1% |
| Gsn | 1.063 | 6.12E-52 | 77.7% | 28.6% |
| S100a6 | 1.066 | 3.38E-51 | 76.2% | 24.8% |
| Ifitm2 | 0.928 | 1.19E-47 | 95.3% | 53.3% |
| Syngr2 | 0.887 | 3.26E-47 | 89.8% | 43.8% |
| Ifitm1 | 1.537 | 8.14E-47 | 39.5% | 4.8% |
| Rpl18a | 0.576 | 7.12E-46 | 100.0% | 96.1% |
| Vasp | 0.914 | 4.23E-45 | 89.1% | 49.3% |
| Rps6 | 0.562 | 1.13E-44 | 100.0% | 95.4% |
| Ifitm3 | 0.700 | 1.27E-44 | 96.9% | 53.0% |
| Klrk1 | 0.969 | 1.75E-44 | 35.2% | 3.5% |
| Rps27rt | 0.629 | 1.42E-42 | 99.6% | 85.1% |
| Rpl13 | 0.485 | 3.39E-42 | 100.0% | 97.5% |
| Rps19 | 0.520 | 5.04E-42 | 100.0% | 96.9% |
| Il1b | 0.813 | 7.86E-42 | 96.1% | 53.0% |

**Supplemental Table 2.** Top 30 differentially expressed genes for cells in each cluster (defined in Figure 3C), listed in ascending order based on adjusted p-value. Differentially expressed genes were identified using the FindMarkers function in Seurat, using a Wilcoxon rank sum test followed by Bonferroni correction. For each cluster, all cells within that cluster were compared to all other cells outside of the cluster. The log_2_FC expression indicates the log_2_ fold-change of the average increase in expression in cells in that cluster compared to the average expression across all other cells. Genes were only considered if they were expressed in at least 25% of the cells within that cluster. Also shown are the percent of cells within and outside of the cluster that express that gene.

A. Microglia

| **Cluster identity** | **Gene symbol** | **Log_2_FC expression** | **Adjusted p-value** | **Cells expressing gene in Cluster (%)** | **All other cells expressing gene (%)** |
| --- | --- | --- | --- | --- | --- |
| **Resting microglia (#1)** | P2ry12 | 2.799 | 7.38e-135 | 97.0% | 13.5% |
|  | Cst3 | 2.297 | 7.70e-84 | 100.0% | 97.7% |
|  | Rhob | 2.081 | 1.39e-83 | 92.2% | 26.0% |
|  | Cd81 | 2.004 | 4.39e-81 | 100.0% | 64.2% |
|  | Tmem119 | 2.026 | 1.72e-79 | 85.5% | 21.2% |
|  | Hexb | 1.689 | 1.10e-78 | 100.0% | 90.3% |
|  | Klf2 | 2.426 | 1.89e-78 | 99.4% | 46.1% |
|  | Gpr34 | 1.900 | 3.14e-78 | 76.5% | 12.8% |
|  | Junb | 1.765 | 7.28e-77 | 100.0% | 88.2% |
|  | Ubc | 1.650 | 5.01e-74 | 99.4% | 89.0% |
|  | Fos | 2.078 | 7.56e-69 | 99.4% | 79.6% |
|  | Sparc | 1.631 | 1.30e-68 | 78.3% | 16.0% |
|  | Jun | 2.396 | 1.64e-68 | 98.2% | 66.3% |
|  | Siglech | 1.508 | 2.54e-68 | 50.0% | 3.9% |
|  | Jund | 1.680 | 1.32e-67 | 98.8% | 75.7% |
|  | Adrb2 | 2.315 | 7.59e-67 | 73.5% | 15.8% |
|  | Egr1 | 1.838 | 1.64e-65 | 94.0% | 39.7% |
|  | Sepp1 | 1.754 | 2.56e-59 | 93.4% | 42.7% |
|  | Ccl3 | 2.225 | 8.18e-59 | 95.8% | 67.4% |
|  | Ctss | 1.038 | 2.05e-58 | 100.0% | 96.7% |
|  | Ltc4s | 1.329 | 7.10e-58 | 61.4% | 10.0% |
|  | Selplg | 1.814 | 9.49e-58 | 92.8% | 52.1% |
|  | Il1a | 1.858 | 1.21e-57 | 77.1% | 21.7% |
|  | C1qc | 1.221 | 3.54e-57 | 100.0% | 79.1% |
|  | Dusp1 | 1.483 | 2.59e-53 | 99.4% | 81.6% |
|  | C1qb | 1.094 | 9.33e-50 | 100.0% | 84.3% |
|  | Ccl4 | 2.076 | 2.92e-49 | 96.4% | 73.8% |
|  | Ier2 | 1.486 | 2.96e-49 | 98.8% | 73.9% |
|  | Serpine2 | 1.323 | 7.97e-48 | 86.1% | 32.8% |
|  | Serinc3 | 1.238 | 1.71e-47 | 95.2% | 61.4% |
| **Activated microglia (A) (#2)** | Atp6v0d2 | 1.085 | 6.27e-83 | 46.4% | 0.4% |
|  | Mcoln3 | 0.727 | 1.20e-74 | 42.9% | 0.4% |
|  | Cdo1 | 1.622 | 5.29e-63 | 57.1% | 1.6% |
|  | Cd5l | 2.902 | 2.01e-55 | 71.4% | 3.6% |
|  | Gpx3 | 3.029 | 2.03e-54 | 85.7% | 6.1% |
|  | Gm1673 | 1.368 | 7.73e-49 | 71.4% | 4.0% |
|  | Ahnak2 | 0.393 | 2.12e-30 | 28.6% | 0.7% |
|  | Pianp | 0.658 | 2.64e-30 | 42.9% | 2.1% |
|  | Cst7 | 2.210 | 5.78e-28 | 89.3% | 13.9% |
|  | Apoc4 | 0.834 | 4.27e-27 | 42.9% | 2.5% |
|  | Mgll | 0.496 | 2.27e-26 | 28.6% | 0.9% |
|  | Gpnmb | 3.022 | 1.10e-24 | 96.4% | 22.1% |
|  | Chst2 | 0.276 | 2.24e-22 | 35.7% | 1.9% |
|  | Fabp3 | 0.827 | 2.14e-20 | 60.7% | 6.8% |
|  | Igf1 | 1.282 | 2.30e-20 | 75.0% | 11.2% |
|  | Ttc3 | 0.627 | 1.02e-19 | 35.7% | 2.3% |
|  | Pld3 | 2.354 | 4.11e-18 | 92.9% | 29.8% |
|  | Timp2 | 2.396 | 6.44e-18 | 100.0% | 38.8% |
|  | Syngr1 | 2.245 | 3.63e-17 | 100.0% | 39.3% |
|  | Dpp7 | 0.930 | 9.54e-17 | 53.6% | 6.4% |
|  | Glb1 | 0.810 | 5.98e-16 | 60.7% | 8.3% |
|  | Creg1 | 1.856 | 1.05e-15 | 100.0% | 50.1% |
|  | Lag3 | 1.027 | 1.07e-15 | 85.7% | 17.0% |
|  | Ctla2a | 0.571 | 1.14e-15 | 35.7% | 2.8% |
|  | Cd63 | 1.965 | 1.76e-14 | 100.0% | 63.0% |
|  | Fabp5 | 1.298 | 3.12e-13 | 96.4% | 28.0% |
|  | Lyz1 | 0.773 | 3.93e-13 | 67.9% | 11.8% |
|  | Apoe | 2.114 | 5.70e-13 | 100.0% | 99.3% |
|  | Hpse | 0.675 | 1.50e-12 | 39.3% | 4.3% |
|  | Pon3 | 0.990 | 1.64e-12 | 60.7% | 10.9% |
| **Activated microglia (B) (#3)** | Tmsb4x | 0.741 | 3.17e-30 | 100.0% | 99.8% |
|  | Gapdh | 0.682 | 3.97e-14 | 92.2% | 82.6% |
|  | Ctsd | 0.974 | 1.95e-13 | 90.7% | 89.8% |
|  | Cd63 | 0.846 | 6.59e-13 | 83.7% | 61.4% |
|  | Fth1 | 0.636 | 3.64e-12 | 100.0% | 99.7% |
|  | C1qa | 0.634 | 1.27e-11 | 97.7% | 78.3% |
|  | Gm10116 | 0.571 | 3.61e-11 | 100.0% | 99.6% |
|  | Ctsz | 0.609 | 7.94e-11 | 95.3% | 94.1% |
|  | Cd81 | 0.493 | 1.16e-10 | 90.7% | 66.7% |
|  | Pkm | 0.816 | 3.31e-10 | 84.5% | 79.8% |
|  | Ftl1 | 0.521 | 4.04e-10 | 100.0% | 99.9% |
|  | C1qc | 0.571 | 1.63e-09 | 95.3% | 80.5% |
|  | Gatm | 0.754 | 3.41e-08 | 79.8% | 65.2% |
|  | C1qb | 0.548 | 3.87e-08 | 96.9% | 85.3% |
|  | Mif | 0.861 | 1.16e-07 | 69.8% | 58.1% |
|  | Fcrls | 0.898 | 1.95e-07 | 54.3% | 31.4% |
|  | Aldoa | 0.678 | 4.38e-06 | 85.3% | 83.9% |
|  | Serpine2 | 0.641 | 8.57e-06 | 62.8% | 37.8% |
|  | Prdx1 | 0.512 | 5.76e-05 | 89.9% | 83.2% |
|  | Tuba1b | 0.541 | 6.28e-05 | 79.1% | 65.5% |
|  | Mt1 | 1.024 | 6.34e-05 | 64.3% | 51.5% |
|  | Fcer1g | 0.446 | 0.000109933 | 97.7% | 94.1% |
|  | Ctsb | 0.486 | 0.000215108 | 97.7% | 96.6% |
|  | Cd9 | 0.553 | 0.000328161 | 88.4% | 82.8% |
|  | Cstb | 0.512 | 0.000611186 | 77.5% | 65.7% |
|  | Rpl41 | 0.334 | 0.001352796 | 100.0% | 98.1% |
|  | Ppia | 0.294 | 0.001614957 | 99.2% | 96.5% |
|  | Aif1 | 0.616 | 0.001615488 | 78.3% | 70.4% |
|  | Syngr1 | 0.568 | 0.008185932 | 55.0% | 39.0% |
|  | Hexb | 0.315 | 0.018391534 | 93.8% | 91.5% |
| **Activated microglia, proliferating (#4)** | 2810417H13Rik | 1.497 | 7.16e-51 | 64.6% | 10.1% |
|  | Birc5 | 1.450 | 1.49e-45 | 54.0% | 7.2% |
|  | Stmn1 | 1.409 | 7.66e-41 | 77.9% | 22.8% |
|  | Ube2c | 1.555 | 1.68e-33 | 42.5% | 6.0% |
|  | Cdk1 | 1.381 | 8.85e-32 | 46.9% | 8.4% |
|  | Tuba1b | 1.161 | 6.65e-31 | 92.9% | 64.2% |
|  | Tubb5 | 1.280 | 4.00e-30 | 92.0% | 56.7% |
|  | Tmsb4x | 0.832 | 2.50e-27 | 98.2% | 100.0% |
|  | Pbk | 1.536 | 4.86e-26 | 32.7% | 4.4% |
|  | Rhoc | 1.058 | 1.17e-22 | 57.5% | 16.9% |
|  | Cdca3 | 0.984 | 4.49e-21 | 29.2% | 4.2% |
|  | Gapdh | 0.808 | 1.83e-20 | 95.6% | 82.4% |
|  | Ccnb1 | 0.811 | 6.65e-19 | 27.4% | 4.0% |
|  | Pkm | 0.802 | 1.33e-17 | 94.7% | 78.7% |
|  | Ppia | 0.576 | 2.22e-17 | 100.0% | 96.4% |
|  | Gatm | 1.167 | 4.32e-17 | 86.7% | 64.7% |
|  | Cenpa | 0.821 | 6.43e-17 | 32.7% | 6.4% |
|  | Cdca8 | 0.980 | 6.40e-15 | 26.5% | 4.7% |
|  | Ran | 0.838 | 2.34e-14 | 85.8% | 66.5% |
|  | Pttg1 | 0.967 | 4.87e-14 | 37.2% | 9.7% |
|  | Hmgn1 | 0.966 | 1.05e-13 | 65.5% | 33.2% |
|  | H2afz | 0.653 | 3.23e-13 | 93.8% | 86.8% |
|  | Ranbp1 | 0.761 | 3.41e-13 | 89.4% | 69.1% |
|  | Tubb4b | 1.139 | 1.06e-12 | 46.0% | 16.3% |
|  | Prdx1 | 0.663 | 1.09e-12 | 95.6% | 82.7% |
|  | Kpna2 | 0.897 | 2.96e-12 | 43.4% | 15.0% |
|  | Fth1 | 0.579 | 8.82e-12 | 100.0% | 99.7% |
|  | Tyms | 0.891 | 9.43e-12 | 33.6% | 9.2% |
|  | Tacc3 | 0.864 | 9.87e-12 | 28.3% | 6.3% |
|  | Spc25 | 0.882 | 1.98e-11 | 26.5% | 5.9% |
| **Activated microglia (C) (#5)** | Golga3 | 1.092 | 7.30e-20 | 44.9% | 6.2% |
|  | Ch25h | 2.020 | 1.77e-18 | 51.0% | 8.9% |
|  | Slc31a2 | 1.228 | 8.56e-17 | 51.0% | 9.4% |
|  | Pola2 | 1.117 | 4.27e-16 | 63.3% | 15.4% |
|  | Bcl2l1 | 0.847 | 2.17e-14 | 32.7% | 4.2% |
|  | Agl | 1.536 | 9.94e-14 | 40.8% | 7.0% |
|  | Pmp22 | 0.950 | 7.47e-12 | 65.3% | 18.5% |
|  | RP23-4H17.3 | 0.945 | 1.11e-11 | 44.9% | 9.9% |
|  | Bhlhe41 | 0.617 | 2.43e-11 | 28.6% | 3.8% |
|  | Hist1h1c | 1.082 | 3.77e-11 | 36.7% | 6.5% |
|  | Chst1 | 0.550 | 8.06e-11 | 30.6% | 4.5% |
|  | Bag5 | 0.909 | 1.13e-10 | 34.7% | 6.0% |
|  | Tmem245 | 0.940 | 1.47e-10 | 46.9% | 11.5% |
|  | Agpat3 | 1.418 | 1.57e-10 | 28.6% | 4.2% |
|  | Trmt2a | 0.415 | 1.66e-10 | 40.8% | 7.8% |
|  | Gm13075 | 0.564 | 2.14e-10 | 36.7% | 6.9% |
|  | Usp2 | 0.537 | 4.09e-10 | 38.8% | 7.3% |
|  | Timm10 | 0.558 | 8.88e-10 | 32.7% | 5.4% |
|  | Mid1ip1 | 0.948 | 3.46e-09 | 40.8% | 8.8% |
|  | Srpk1 | 0.706 | 4.22e-09 | 49.0% | 13.1% |
|  | Malat1 | 1.026 | 7.82e-09 | 98.0% | 81.6% |
|  | Lpl | 1.631 | 1.93e-08 | 55.1% | 18.4% |
|  | Rab7b | 1.163 | 2.03e-08 | 46.9% | 13.2% |
|  | Fam212a | 0.557 | 3.08e-08 | 26.5% | 4.1% |
|  | Znrf1 | 0.993 | 4.43e-08 | 28.6% | 4.9% |
|  | Adamts1 | 0.573 | 4.66e-08 | 26.5% | 4.2% |
|  | Xist | 1.053 | 5.72e-08 | 71.4% | 30.5% |
|  | Dnmt3a | 0.678 | 6.10e-08 | 26.5% | 4.4% |
|  | Tns3 | 0.871 | 1.12e-07 | 34.7% | 7.4% |
|  | Slc2a1 | 0.335 | 2.74e-07 | 32.7% | 6.5% |

B. Monocytes and Macrophages

| **Cluster identity** | **Gene symbol** | **Log_2_FC expression** | **Adjusted p-value** | **Cells expressing gene in Cluster (%)** | **All other cells expressing gene (%)** |
| --- | --- | --- | --- | --- | --- |
| **Circulating monocytes (#6)** | Serpinb10 | 1.042 | 1.61e-49 | 26.8% | 0.3% |
|  | Plac8 | 2.296 | 1.65e-28 | 87.5% | 27.5% |
|  | Ifitm6 | 1.810 | 4.58e-28 | 57.1% | 8.9% |
|  | Treml4 | 1.548 | 5.31e-28 | 37.5% | 3.3% |
|  | Tppp3 | 1.785 | 9.20e-28 | 41.1% | 4.1% |
|  | Ly6i | 1.675 | 3.13e-27 | 26.8% | 1.4% |
|  | Pglyrp1 | 1.690 | 2.20e-26 | 30.4% | 2.1% |
|  | Cebpb | 2.114 | 3.98e-26 | 98.2% | 79.2% |
|  | Mgst1 | 2.121 | 5.79e-25 | 51.8% | 8.1% |
|  | Hp | 1.896 | 9.82e-24 | 46.4% | 6.6% |
|  | Ifitm3 | 1.672 | 2.28e-23 | 98.2% | 61.1% |
|  | Clec4a3 | 1.682 | 1.50e-20 | 64.3% | 15.6% |
|  | H2-D1 | 0.944 | 1.50e-18 | 100.0% | 94.1% |
|  | Metrnl | 1.890 | 6.41e-17 | 75.0% | 29.0% |
|  | Lyz2 | 1.363 | 3.00e-16 | 100.0% | 95.8% |
|  | Prr13 | 1.601 | 2.45e-15 | 89.3% | 46.1% |
|  | Ifitm2 | 1.168 | 4.09e-15 | 92.9% | 61.3% |
|  | Msrb1 | 1.448 | 2.82e-14 | 82.1% | 40.6% |
|  | Samhd1 | 1.564 | 5.50e-14 | 67.9% | 24.9% |
|  | Gpx1 | 1.092 | 6.02e-14 | 98.2% | 86.9% |
|  | Sirpb1c | 1.190 | 1.22e-12 | 55.4% | 16.7% |
|  | Alox5ap | 1.334 | 5.83e-12 | 87.5% | 61.4% |
|  | Clec4a1 | 1.163 | 6.41e-12 | 44.6% | 10.5% |
|  | Eno3 | 1.508 | 1.49e-10 | 26.8% | 4.1% |
|  | Dusp1 | 0.803 | 2.98e-10 | 98.2% | 83.5% |
|  | Plaur | 1.307 | 1.58e-09 | 76.8% | 43.5% |
|  | Ly6c2 | 1.615 | 1.62e-09 | 33.9% | 7.2% |
|  | Adgre5 | 1.366 | 3.63e-09 | 35.7% | 8.2% |
|  | Gngt2 | 1.716 | 1.05e-08 | 75.0% | 48.5% |
|  | Ly6e | 0.892 | 2.51e-08 | 92.9% | 66.2% |
| **Classical Monocytes (#7)** | Acp5 | 1.658 | 6.45e-57 | 86.1% | 25.4% |
|  | Apoc2 | 0.467 | 1.06e-55 | 41.6% | 2.5% |
|  | F10 | 0.968 | 1.38e-54 | 39.4% | 2.3% |
|  | Lyz2 | 1.632 | 9.87e-51 | 100.0% | 95.4% |
|  | Lgals3 | 1.326 | 3.26e-43 | 99.3% | 69.0% |
|  | Ms4a7 | 1.193 | 1.91e-42 | 73.0% | 18.3% |
|  | 4930430E12Rik | 0.703 | 2.00e-42 | 56.2% | 9.6% |
|  | Apoe | 1.187 | 6.93e-42 | 100.0% | 99.2% |
|  | Ctsb | 1.033 | 4.63e-40 | 100.0% | 96.3% |
|  | Lgals1 | 1.100 | 6.69e-40 | 99.3% | 56.0% |
|  | Gm10116 | 0.828 | 9.84e-40 | 100.0% | 99.6% |
|  | Spp1 | 1.810 | 8.06e-38 | 100.0% | 87.7% |
|  | Nr1h3 | 0.764 | 5.69e-37 | 35.8% | 3.6% |
|  | Ftl1 | 0.766 | 2.02e-36 | 100.0% | 99.9% |
|  | Pla2g7 | 1.057 | 2.03e-35 | 54.7% | 11.6% |
|  | Msrb1 | 0.841 | 5.60e-35 | 92.0% | 35.9% |
|  | Cyba | 0.761 | 9.28e-35 | 100.0% | 89.0% |
|  | Arg1 | 1.805 | 1.29e-34 | 48.2% | 9.4% |
|  | Lyz1 | 0.484 | 7.67e-30 | 46.7% | 8.5% |
|  | Cstb | 0.990 | 8.98e-30 | 95.6% | 63.1% |
|  | Fn1 | 0.882 | 1.46e-29 | 67.9% | 20.3% |
|  | Cyp4f18 | 0.926 | 1.43e-27 | 82.5% | 35.0% |
|  | Uap1l1 | 0.598 | 3.24e-27 | 67.9% | 20.5% |
|  | Chil3 | 0.759 | 8.13e-27 | 43.1% | 8.2% |
|  | Clec4e | 0.618 | 1.09e-26 | 53.3% | 12.9% |
|  | Tnfsf12 | 0.571 | 1.59e-26 | 38.7% | 6.5% |
|  | Tgfbi | 0.842 | 4.28e-26 | 89.8% | 41.2% |
|  | Cd24a | 0.822 | 4.42e-26 | 46.7% | 10.3% |
|  | Gpnmb | 0.643 | 1.67e-24 | 59.1% | 19.0% |
|  | Emp1 | 0.447 | 4.50e-24 | 46.7% | 10.4% |
| **Inflammatory Monocytes (#8)** | Il1b | 1.217 | 3.01e-40 | 94.6% | 57.2% |
|  | Tgfbi | 1.023 | 1.88e-39 | 89.9% | 39.6% |
|  | Ctsc | 0.914 | 1.56e-35 | 98.2% | 85.7% |
|  | Cxcl2 | 1.328 | 2.19e-35 | 96.4% | 65.5% |
|  | Cyp4f18 | 0.966 | 3.16e-31 | 79.8% | 33.9% |
|  | Ccr2 | 0.835 | 3.30e-29 | 66.7% | 24.1% |
|  | Ms4a6c | 0.772 | 3.95e-29 | 92.9% | 50.6% |
|  | Fn1 | 0.895 | 1.45e-28 | 61.3% | 19.9% |
|  | Ms4a6d | 0.863 | 1.62e-28 | 83.9% | 41.2% |
|  | Sirpb1c | 0.700 | 8.49e-27 | 50.6% | 13.0% |
|  | Klra2 | 0.893 | 9.34e-27 | 35.7% | 6.4% |
|  | Clec4a2 | 0.769 | 2.93e-26 | 51.2% | 13.7% |
|  | Prdx5 | 0.793 | 1.48e-25 | 94.6% | 71.0% |
|  | Ms4a4c | 0.863 | 5.81e-25 | 59.5% | 19.4% |
|  | Cebpb | 0.525 | 4.61e-23 | 98.2% | 77.0% |
|  | Lst1 | 0.738 | 5.40e-23 | 59.5% | 20.3% |
|  | Ifi27l2a | 1.034 | 8.00e-23 | 78.0% | 39.7% |
|  | Clec12a | 1.149 | 1.24e-22 | 50.6% | 15.6% |
|  | Ccr1 | 0.804 | 1.29e-22 | 66.1% | 27.8% |
|  | Ccl7 | 1.641 | 3.65e-21 | 61.3% | 27.1% |
|  | Apoe | 0.485 | 3.35e-19 | 100.0% | 99.2% |
|  | Cd14 | 0.694 | 8.49e-18 | 95.2% | 78.3% |
|  | Fcer1g | 0.473 | 8.80e-18 | 100.0% | 93.5% |
|  | Ly6e | 0.689 | 3.29e-16 | 91.1% | 63.4% |
|  | Ifitm3 | 0.575 | 6.90e-16 | 92.9% | 57.7% |
|  | Ms4a6b | 0.750 | 1.77e-15 | 63.1% | 30.5% |
|  | Sirpb1b | 0.367 | 7.93e-15 | 36.9% | 10.4% |
|  | Lgals1 | 0.456 | 1.20e-14 | 89.9% | 56.3% |
|  | Pilra | 0.613 | 7.04e-14 | 47.0% | 18.0% |
|  | Zbp1 | 0.703 | 1.08e-13 | 34.5% | 10.4% |
| **Inflammatory macrophages (#9)** | H2-Aa | 1.993 | 4.96e-97 | 100.0% | 64.3% |
|  | Cd74 | 1.906 | 8.40e-97 | 100.0% | 81.5% |
|  | H2-Ab1 | 2.014 | 6.63e-96 | 100.0% | 73.1% |
|  | H2-Eb1 | 2.024 | 5.13e-95 | 100.0% | 58.6% |
|  | H2-DMb2 | 1.930 | 7.99e-94 | 88.8% | 24.2% |
|  | Napsa | 1.723 | 5.24e-81 | 88.3% | 27.2% |
|  | H2-DMb1 | 1.451 | 1.60e-72 | 98.1% | 48.0% |
|  | H2-Oa | 1.641 | 1.39e-68 | 56.8% | 7.1% |
|  | Plbd1 | 1.378 | 8.78e-66 | 91.3% | 34.1% |
|  | Mdh2 | 1.459 | 3.76e-64 | 96.1% | 59.9% |
|  | H2-DMa | 1.211 | 4.24e-62 | 99.0% | 58.9% |
|  | Itgb7 | 1.083 | 3.65e-59 | 53.9% | 7.3% |
|  | Cfp | 1.317 | 7.22e-57 | 84.0% | 30.6% |
|  | Lsp1 | 1.007 | 1.73e-50 | 88.3% | 38.0% |
|  | Crip1 | 1.047 | 5.86e-50 | 96.6% | 57.0% |
|  | Bhlhe40 | 1.226 | 6.69e-50 | 80.1% | 31.9% |
|  | Il1r2 | 1.299 | 7.34e-48 | 57.3% | 13.0% |
|  | Tmsb10 | 0.860 | 6.91e-47 | 99.0% | 78.0% |
|  | S100a11 | 1.282 | 7.26e-45 | 76.7% | 28.2% |
|  | Rps27rt | 0.697 | 2.71e-44 | 99.5% | 85.9% |
|  | Ifitm2 | 0.968 | 7.75e-44 | 96.1% | 55.4% |
|  | Rpl18a | 0.612 | 1.98e-42 | 100.0% | 96.3% |
|  | Syngr2 | 0.964 | 2.85e-42 | 89.3% | 46.4% |
|  | Ifitm1 | 1.512 | 7.60e-42 | 41.7% | 6.2% |
|  | Ifitm3 | 0.774 | 6.30e-41 | 96.6% | 55.4% |
|  | Rpl13 | 0.514 | 4.04e-40 | 100.0% | 97.6% |
|  | S100a6 | 1.063 | 3.42e-39 | 74.3% | 28.0% |
|  | Rps27 | 0.684 | 3.61e-39 | 99.5% | 90.4% |
|  | Rps9 | 0.624 | 4.10e-39 | 100.0% | 93.6% |
|  | Epcam | 0.975 | 6.08e-39 | 34.0% | 3.7% |
| **Macrophages, proliferating (#10)** | Hist1h2ap | 0.778 | 1.13e-41 | 41.9% | 1.9% |
|  | Rrm2 | 1.398 | 3.50e-39 | 72.1% | 7.9% |
|  | 2810417H13Rik | 1.704 | 2.23e-36 | 83.7% | 12.9% |
|  | Asf1b | 1.069 | 2.91e-34 | 67.4% | 7.7% |
|  | Spc24 | 1.034 | 1.01e-31 | 65.1% | 7.8% |
|  | Dut | 0.966 | 4.92e-31 | 79.1% | 11.5% |
|  | Cenpm | 0.744 | 1.01e-30 | 44.2% | 3.2% |
|  | Tk1 | 1.196 | 2.91e-28 | 60.5% | 7.2% |
|  | Top2a | 0.631 | 4.24e-28 | 34.9% | 2.0% |
|  | H2afx | 1.455 | 2.03e-27 | 74.4% | 12.7% |
|  | Mcm7 | 0.877 | 9.64e-27 | 60.5% | 7.7% |
|  | Stmn1 | 1.469 | 3.19e-25 | 97.7% | 25.6% |
|  | Smc2 | 0.687 | 7.22e-25 | 39.5% | 3.2% |
|  | Cks1b | 1.006 | 1.33e-24 | 86.0% | 17.4% |
|  | Cdca3 | 0.557 | 3.14e-24 | 48.8% | 5.0% |
|  | Nusap1 | 0.807 | 7.48e-24 | 41.9% | 3.8% |
|  | Birc5 | 1.106 | 8.16e-24 | 65.1% | 9.8% |
|  | Cks1brt | 0.509 | 1.46e-23 | 65.1% | 9.3% |
|  | Dcstamp | 0.644 | 4.47e-23 | 37.2% | 3.0% |
|  | Ccna2 | 0.671 | 1.24e-22 | 44.2% | 4.3% |
|  | Haus4 | 0.597 | 1.26e-22 | 48.8% | 5.5% |
|  | Tyms | 0.831 | 9.56e-22 | 62.8% | 9.6% |
|  | Hmgb2 | 1.585 | 9.69e-22 | 100.0% | 43.3% |
|  | Plp2 | 0.619 | 1.80e-20 | 74.4% | 13.9% |
|  | Racgap1 | 0.582 | 2.62e-20 | 44.2% | 4.9% |
|  | Qpct | 1.183 | 4.70e-20 | 88.4% | 22.8% |
|  | Cenph | 0.472 | 5.81e-20 | 25.6% | 1.5% |
|  | Ptma | 1.006 | 6.48e-20 | 100.0% | 96.4% |
|  | Apitd1 | 0.439 | 1.18e-19 | 30.2% | 2.2% |
|  | Rfc5 | 0.652 | 2.21e-19 | 58.1% | 9.1% |

C. Unidentified clusters:

| **Cluster identity** | **Gene symbol** | **Log_2_FC expression** | **Adjusted p-value** | **Cells expressing gene in Cluster (%)** | **All other cells expressing gene (%)** |
| --- | --- | --- | --- | --- | --- |
| **Unidentified 1 (#11)** | Esam | 2.640 | 4.80e-163 | 90.0% | 0.3% |
|  | Foxq1 | 1.946 | 4.32e-154 | 80.0% | 0.2% |
|  | Tmem252 | 2.637 | 2.81e-150 | 90.0% | 0.4% |
|  | Sox17 | 2.722 | 3.24e-150 | 90.0% | 0.4% |
|  | S100a16 | 2.488 | 6.74e-130 | 90.0% | 0.5% |
|  | Pcp4l1 | 2.044 | 2.15e-117 | 70.0% | 0.3% |
|  | Egfl7 | 3.115 | 2.08e-108 | 90.0% | 0.8% |
|  | Ramp2 | 1.793 | 2.50e-106 | 70.0% | 0.4% |
|  | Sparcl1 | 2.832 | 2.93e-97 | 70.0% | 0.4% |
|  | Ptn | 2.417 | 5.94e-95 | 60.0% | 0.3% |
|  | Itm2a | 3.777 | 2.74e-93 | 100.0% | 1.4% |
|  | Slc9a3r2 | 1.869 | 2.46e-92 | 90.0% | 1.1% |
|  | AU021092 | 1.774 | 3.59e-85 | 60.0% | 0.4% |
|  | Ly6c1 | 3.661 | 3.07e-78 | 100.0% | 1.9% |
|  | Abcb1a | 1.566 | 1.68e-77 | 60.0% | 0.4% |
|  | Stra6 | 1.228 | 9.71e-77 | 60.0% | 0.4% |
|  | Flt1 | 2.399 | 2.89e-68 | 70.0% | 0.9% |
|  | Cyr61 | 2.590 | 3.42e-68 | 70.0% | 0.9% |
|  | Cldn5 | 4.314 | 2.04e-67 | 100.0% | 2.3% |
|  | Crip2 | 2.607 | 5.69e-63 | 80.0% | 1.4% |
|  | Hspb1 | 1.783 | 5.36e-60 | 70.0% | 1.1% |
|  | Ctla2a | 3.308 | 6.67e-59 | 100.0% | 2.8% |
|  | Maoa | 0.944 | 2.54e-55 | 60.0% | 0.8% |
|  | Lsr | 1.839 | 2.88e-51 | 90.0% | 2.5% |
|  | Igfbp7 | 4.589 | 1.53e-49 | 100.0% | 3.5% |
|  | Ly6a | 3.481 | 4.25e-49 | 100.0% | 3.5% |
|  | Gsta4 | 1.116 | 3.35e-40 | 40.0% | 0.4% |
|  | Vwa1 | 1.995 | 1.36e-37 | 70.0% | 2.1% |
|  | Vamp5 | 1.558 | 7.80e-37 | 60.0% | 1.4% |
|  | Pir | 1.552 | 2.86e-36 | 40.0% | 0.5% |
| **Unidentified 2 (#12)** | Ccr7 | 3.765 | 5.60e-82 | 100.0% | 3.4% |
|  | Cacnb3 | 1.882 | 1.35e-72 | 52.6% | 0.6% |
|  | Tbc1d4 | 1.816 | 4.12e-51 | 63.2% | 2.0% |
|  | Spint2 | 2.528 | 7.90e-44 | 84.2% | 5.0% |
|  | Serpinb6b | 2.116 | 1.97e-41 | 47.4% | 1.3% |
|  | Cd200 | 1.975 | 1.70e-39 | 63.2% | 2.8% |
|  | Birc2 | 1.921 | 1.54e-25 | 63.2% | 4.7% |
|  | Zmynd15 | 2.519 | 6.50e-22 | 63.2% | 5.7% |
|  | Fscn1 | 2.769 | 6.79e-22 | 100.0% | 18.1% |
|  | Relb | 3.105 | 2.99e-20 | 68.4% | 7.2% |
|  | Gypc | 0.953 | 1.70e-19 | 26.3% | 0.8% |
|  | Ccl5 | 3.857 | 1.93e-19 | 94.7% | 18.3% |
|  | Socs2 | 1.540 | 6.70e-19 | 36.8% | 1.9% |
|  | Hmgn3 | 1.494 | 2.24e-18 | 57.9% | 5.2% |
|  | Cd1d1 | 1.563 | 3.65e-18 | 42.1% | 2.6% |
|  | Ramp3 | 1.292 | 2.36e-17 | 31.6% | 1.4% |
|  | Vsig10 | 1.077 | 1.26e-16 | 26.3% | 1.0% |
|  | Tmem123 | 3.162 | 1.38e-16 | 100.0% | 27.5% |
|  | Itga4 | 1.755 | 2.29e-15 | 52.6% | 5.2% |
|  | Gnb4 | 1.164 | 1.34e-14 | 36.8% | 2.4% |
|  | Cnn2 | 1.773 | 1.00e-13 | 94.7% | 21.5% |
|  | Fabp5 | 2.049 | 3.67e-13 | 100.0% | 28.5% |
|  | Gbp2 | 1.222 | 6.28e-13 | 36.8% | 2.7% |
|  | Pcgf5 | 1.495 | 7.80e-11 | 47.4% | 5.3% |
|  | Il4i1 | 1.659 | 1.20e-10 | 31.6% | 2.4% |
|  | Lsp1 | 2.279 | 1.46e-10 | 100.0% | 46.3% |
|  | Tspan3 | 2.754 | 2.15e-10 | 73.7% | 16.7% |
|  | Fam177a | 1.655 | 2.25e-10 | 63.2% | 10.7% |
|  | Supt4a | 1.413 | 1.66e-09 | 94.7% | 30.2% |
|  | Bhlhe40 | 1.666 | 2.96e-09 | 100.0% | 39.7% |
